# Estimated COVID-19 Cases and Hospitalizations Averted by Case Investigation and Contact Tracing in the US

**DOI:** 10.1001/jamanetworkopen.2022.4042

**Published:** 2022-03-25

**Authors:** Gabriel Rainisch, Seonghye Jeon, Danielle Pappas, Kimberly D. Spencer, Leah S. Fischer, Bishwa B. Adhikari, Melanie M. Taylor, Bradford Greening, Patrick K. Moonan, John E. Oeltmann, Emily B. Kahn, Michael L. Washington, Martin I. Meltzer

**Affiliations:** 1 COVID-19 Response Team, Centers for Disease Control and Prevention, Atlanta, Georgia; 2 Epidemiology and Laboratory Capacity Program, Centers for Disease Control and Prevention, Atlanta, Georgia

## Abstract

**Importance:**

Evidence of the impact of COVID-19 case investigation and contact tracing (CICT) programs is lacking, but policy makers need this evidence to assess the value of such programs.

**Objective:**

To estimate COVID-19 cases and hospitalizations averted nationwide by US states’ CICT programs.

**Design, Setting, and Participants:**

This decision analytical model study used combined data from US CICT programs (eg, proportion of cases interviewed, contacts notified or monitored, and days to case and contact notification) with incidence data to model outcomes of CICT over a 60-day period (November 25, 2020, to January 23, 2021). The study estimated a range of outcomes by varying assumed compliance with isolation and quarantine recommendations. Fifty-nine state and territorial health departments that received federal funding supporting COVID-19 pandemic response activities were eligible for inclusion. Data analysis was performed from July to September 2021.

**Exposure:**

Public health case investigation and contact tracing.

**Main Outcomes and Measures:**

The primary outcomes were numbers of cases and hospitalizations averted and the percentage of cases averted among cases not prevented by vaccination and other nonpharmaceutical interventions.

**Results:**

In total, 22 states and 1 territory reported all measures necessary for the analysis. These 23 jurisdictions covered 42.5% of the US population (approximately 140 million persons), spanned all 4 US Census regions, and reported data that reflected all 59 federally funded CICT programs. This study estimated that 1.11 million cases and 27 231 hospitalizations were averted by CICT programs under a scenario where 80% of interviewed cases and monitored contacts and 30% of notified contacts fully complied with isolation and quarantine guidance, eliminating their contributions to future transmission. As many as 1.36 million cases and 33 527 hospitalizations could have been prevented if all interviewed cases and monitored contacts had entered into and fully complied with isolation and quarantine guidelines upon being interviewed or notified. Across both scenarios and all jurisdictions, CICT averted an estimated median of 21.2% (range, 1.3%-65.8%) of the cases not prevented by vaccination and other nonpharmaceutical interventions.

**Conclusions and Relevance:**

These findings suggest that CICT programs likely had a substantial role in curtailing the pandemic in most jurisdictions during the 2020 to 2021 winter peak. Differences in outcomes across jurisdictions indicate an opportunity to further improve CICT effectiveness. These estimates demonstrate the potential benefits from sustaining and improving these programs.

## Key Points

**Question:**

What are the estimated numbers of COVID-19 cases and hospitalizations averted by case investigation and contact tracing (CICT) programs in the US?

**Findings:**

This decision analytical model study used CICT program data from 23 jurisdictions and estimated that CICT programs averted 1.11 to 1.36 million cases and 27 231 to 33 527 hospitalizations over 60 days during the 2020 to 2021 winter peak of the pandemic. The upper estimate assumes that all interviewed cases and monitored contacts complied with isolation and quarantine guidelines, whereas the lower estimate assumes that fractions of interviewed cases and monitored or notified contacts did so.

**Meaning:**

These findings suggest that CICT programs likely played a critical role in curtailing the pandemic.

## Teaser

This decision analytical model estimates the numbers of COVID-19 cases and hospitalizations averted nationwide by US states’ case investigation and contact tracing programs.

## Introduction

Reducing exposure to persons with communicable diseases through isolation and quarantine are basic tenets of transmission prevention. Public health programs regularly conduct case investigation and contact tracing (CICT) as a means of notifying persons infected with or exposed to communicable diseases and, often, of their need to isolate or quarantine. However, evidence of the role of CICT in mitigating the COVID-19 pandemic thus far is lacking.^1^ We recently showed, using data from 14 US jurisdictions (5 states and 9 local health districts), that CICT programs were estimated to have reduced SARS-CoV-2 transmission by 0.4% to 32%.^2^ Despite these findings, the value of CICT remain controversial.^3,4,5,6^ Some claim that the benefits are limited because of the difficulty in scaling up services during COVID-19 case surges or community reticence to participate in CICT, curtailing meaningful engagement between health departments and cases and their close contacts.^3,6,7^ Between June 2020 and March 2021, the US Centers for Disease Control and Prevention (CDC) distributed more than $40 billion to state, local, and territorial health departments to support COVID-19 response activities, with a notable portion directed toward CICT activities.^5^ A national review of these efforts from November 2020 to March 2021 indicates that 39 000 to 56 000 case investigators and contact tracers (per month) interviewed 9.1 million cases and identified 10.7 million contacts (internal CDC data, A. Schultz, email, March 1, 2022). Given the unprecedented funding and effort surrounding CICT and continuing debate surrounding its value, we sought to present an expanded profile of the outcomes of CICT nationally at its busiest point of the pandemic as of the time of this writing, August 2021.

## Methods

This decision analytical model study follows the Consolidated Health Economic Evaluation Reporting Standards (CHEERS) reporting guideline and was reviewed by CDC for consistency with applicable federal law and CDC policy (eg, 45 CFR part 46, 21 CFR part 56; 42 USC §241(d); 5 USC §552a; 44 USC §3501 et seq).^8^ This study was determined to be public health surveillance; thus, it was not submitted for institutional review board approval, and informed consent was not needed. We used CDC’s COVIDTracer Advanced modeling tool^9^ in combination with data from CICT programs to estimate cases and hospitalizations averted by CICT activities among states and territories funded by CDC’s ELC (Epidemiology and Laboratory Capacity for Prevention and Control of Emerging Infectious Diseases) program. We focused on the 60-day period from November 25, 2020, to January 23, 2021.

### Data

Sixty-four health departments receiving CICT funding report to CDC’s ELC program monthly on the performance of their CICT programs.^5^ We used reported metrics from each jurisdiction to derive its CICT modeled effectiveness measures for the 60-day analysis period: the proportion of cases and contacts who entered into isolation and quarantine because of CICT efforts and the days required to do so (eAppendix 1 and eFigure 1 in Supplement 1). Reported metrics used to calculate CICT modeled effectiveness include the proportions of cases interviewed, contacts notified or monitored, and number of days from testing to case and contact notification. A summary of these data and assumptions used to calculate a range of CICT modeled effectiveness values for each jurisdiction are detailed further in the Range of Estimates section and in eAppendix 1 and eTable 1 in Supplement 1. We limited our analysis to those jurisdictions that reported all the required metrics and passed our data quality checks (eg, the number of contacts identified was greater than or equal to the number cases who provided at least 1 contact, and the number of contacts identified was greater than or equal to the number of contacts notified) (eFigure 2 in Supplement 1).

### Model Use

COVIDTracer Advanced is a deterministic susceptible-exposed-infectious-recovered (SEIR) epidemiological model that illustrates the spread of COVID-19 and impact of interventions in a user-defined population. The tool allows users to attribute reductions in transmission to either CICT or to a combination of vaccination and all other nonpharmaceutical interventions (NPIs), such as face mask policies, large gathering restrictions, and school and business closures (eAppendix 2, eFigure 3, eTable 2, eTable 3, and eTable 4 in Supplement 1). Estimates of reductions in transmission from CICT were obtained by first entering each jurisdiction’s CICT performance into COVIDTracer Advanced. After inputting the CICT performance values for a jurisdiction, we estimated reductions in transmission associated with other NPIs and any inceptive vaccination efforts. We accomplished this by fitting the curve of cumulative cases modeled by COVIDTracer Advanced to the jurisdiction’s reported cases^10^ by altering the percentage reduction in transmission ascribed to vaccine and other NPIs. The value that minimized the deviation (mean-squared error) between the fitted and reported case curves was our estimated effectiveness of vaccine and other NPIs (eTable 5 in Supplement 1). Then we switched off CICT (ie, set CICT effectiveness to 0) while maintaining the estimated effectiveness of vaccine and other NPIs. This simulated what would have happened if CICT had not occurred. Readers can replicate this process with the provided Special Edition version of COVIDTracer Advanced (Supplement 2) and accompanying instructions (eAppendix 3 in Supplement 1).

### Outcome Measures

Estimates of CICT-averted cases were obtained by taking the difference between the model-simulated curve without CICT and jurisdictions’ actual cumulative cases. We also calculated averted hospitalizations by multiplying the estimated number of averted cases by age-stratified infection-to-hospitalization rates (eTable 4 in Supplement 1). In addition to calculating the absolute number of cases and hospitalizations averted by CICT in each jurisdiction, we calculated 2 measures of CICT performance to allow comparison among jurisdictions: (1) averted cases and hospitalizations per 100 000 population, and (2) the proportion of cases or hospitalizations averted by CICT out of the remaining cases, after accounting for vaccination and other NPIs. This latter measure may be interpreted as the number of cases (or hospitalizations) averted by CICT among every 100 cases (or hospitalizations) that were not prevented by vaccination and other NPIs. Finally, we grouped jurisdictions by their US Census Region and compared the group medians of cases averted per 100 000 population to assess whether CICT impact differed among regions.^11^

### Range of Estimates

Jurisdictions did not report the proportions of cases who effectively isolated and contacts who correctly quarantined. Absent compliance data, we generated a range of averted cases and hospitalizations to circumscribe the possible outcomes of CICT. High estimates were calculated by assuming all of the cases a jurisdiction interviewed and all of the contacts it actively monitored fully complied (ie, they isolated 100% of the time over the remaining duration of the recommended isolation or quarantine period) with CDC-recommended isolation and quarantine guidelines (Table 1).^12^ In our high-estimate scenario, we also assumed that contacts who were notified but not actively monitored did not quarantine; that is, we assumed that the cases and contacts CICT programs engaged either fully complied or not at all in this scenario. To produce our low estimates, however, we altered the effect of CICT program’s engagement by lowering the proportions of cases and contacts entering isolation or quarantine on the basis of values derived from the literature (eAppendix 4 in Supplement 1).^7,13,14^ Specifically, we assumed that 80% of cases who completed interviews, 80% of monitored contacts, and 30% of notified contacts (who were not actively monitored) fully complied with isolation and quarantine guidance (Table 1). In our model, and irrespective of these scenarios, infected individuals may transmit to others until interactions with their health department prompt them to isolate or quarantine. Also, both estimates do not include unmeasured changes in behavior from sources of information other than telephone calls or text messages from CICT programs (eg, cases informing their own contacts), as measuring the influence of such factors are beyond our estimation goals.

**Table 1.** Assumed Proportions of Confirmed Cases and Their Contacts Who Effectively Isolated or Quarantined in Each Analysis Scenario

Cases and contacts^a^	Impact scenario, %		Sensitivity analysis, %
	Low	High	
Confirmed cases			
Completed interviews	80	100	100
Did not complete interviews^b^^,^^c^	0	0	0
Contacts			
Were notified and monitored	80	100	100
Were notified but not monitored	30	0	100
Were not notified by their health department^c^	0	0	0

^a^ Each row is a mutually exclusive group of cases or contacts. The sum of each row (or column) does not add up to 100%, because the numbers represent the assumed compliance within each group. Zero percent compliance means none of the cases or contacts in a group isolated or quarantined effectively, and 100% means all of the cases or contacts in a group isolated or quarantined effectively after being interviewed or notified.

^b^ Includes cases who were not reached and those who were reached but who did not agree to be interviewed.

^c^ Compliance was set to 0 for these case-contact group categories because any transmission reductions from quarantine and isolation are not attributable to direct interactions with health department’s case investigation and contact tracing programs staff and, therefore, are outside of the scope of this analysis. Their inclusion here is to help distinguish between the various cases-contacts types.

We also conducted 2 sensitivity analyses. The first evaluated a scenario in which all interviewed cases and all contacts notified of their exposure fully complied with CDC-recommended quarantine guidelines. We chose this aspirational scenario to understand the potential impact of CICT assuming maximum community cooperation. Our second sensitivity analysis evaluated how assuming a background amount of isolation and quarantining would have occurred without direct interactions with health departments would change our findings. That is, instead of setting CICT effectiveness to 0 to simulate an epidemic curve without CICT, we assumed that 20% of interviewed cases would have isolated anyway and that 2.5% of notified contacts would have learned of their exposure via other means (eg, parents receiving notice of their child’s exposure at school) and acted on this knowledge by effectively quarantining. It should be noted that these values represent a hypothetical counterfactual scenario because there are no data on what interviewed cases and notified contacts would have done in the absence of their interactions with their health departments.

### Statistical Analysis

All analyses were executed using the COVIDTracer Advanced model, which was built in Excel for Microsoft 365. Data analysis was performed from July to September 2021.

## Results

Twenty-two US states and 1 territory met our data requirements for inclusion in the analysis (eFigure 2 in Supplement 1). These 23 jurisdictions had a combined population of approximately 140 million persons, covering 42.5% of the entire US population and all 4 US Census regions.^11^ Jurisdictions in our analysis reported metrics (percentage of cases interviewed and contacts notified, and contact notification speed) that were similar to those reported by all 64 federally funded CICT programs (eTable 1 in Supplement 1).

We estimated that CICT averted 1.11 to 1.36 million cases and 27 231 to 33 527 hospitalizations from November 25, 2020, to January 23, 2021, across all 23 jurisdictions analyzed (Figure and eFigure 4 and eTable 5 in Supplement 1). There were 5 269 390 total cases reported across these jurisdictions during the same 60-day period, suggesting that CICT may have reduced the COVID-19 burden by 17% to 21%. The lower estimates assume fractions of interviewed cases and contacts complied with isolation and quarantine guidelines, whereas the upper estimates assume all interviewed cases and monitored contacts did so (Table 1). The median number of estimated cases averted per 100 000 population ranged from 704 (low-impact scenario) to 895 (high-impact scenario). After accounting for vaccination and other NPIs, the median estimate of the percentage of cases averted was 19.1% (range, 1.3%-65.8%) in the low-impact scenario and 23.5% (range, 1.6%-58.7%) in the high-impact scenario, or 21.2% across both scenarios (eTable 5 in Supplement 1). That is, we estimated that CICT averted 1 of every 100 remaining cases not prevented by nascent vaccination efforts and other NPIs in the lowest performing jurisdiction and as many as 66 cases in the highest performing jurisdiction.

**Figure.**
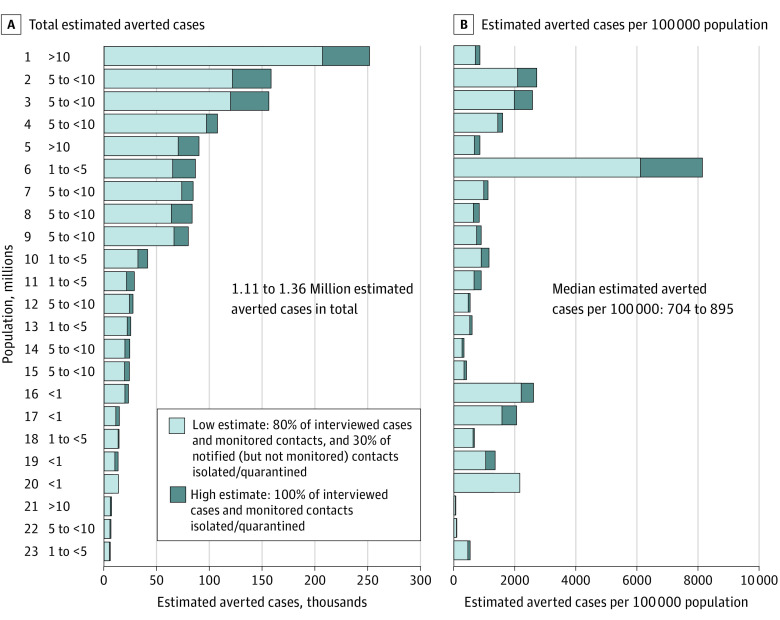
Estimated COVID-19 Cases Averted by Case Investigation and Contact Tracing, by Jurisdiction, November 25, 2020, to January 23, 2021 (60 Days) The numbers 1 through 23 on the left represent the jurisdictions.

On average, the number of estimated cases averted was greater among jurisdictions with larger populations, as shown in panel A of the Figure, with more jurisdictions in the top half having greater populations than those in the bottom half; the median (IQR) population size of jurisdictions was 6.4 million (4.8-9.2 million) in the top half and 3.2 million (1.0-6.0 million) for the bottom half. However, per our estimates, CICT programs in jurisdictions with smaller populations often averted more cases on a per population basis (Figure, panel B). Jurisdictions in the smallest population category (less than 1 million) were estimated to have averted the most cases per population (median, 1714-1875 per 100 000 population), more than twice the overall median (704-895 per 100 000 population). Jurisdictions 5, 6, and 7 were in different population categories (with jurisdiction 5 being 10 times larger than jurisdiction 6), but all 3 jurisdictions were estimated to have averted approximately 87 000 cases under our high-impact scenario (Figure, panel A). Jurisdiction 6’s CICT program is also notable for averting the most estimated cases per 100 000. This result reflects the jurisdiction’s success at interviewing cases (79% interviewed and >50% named at least 1 contact) and being among the fastest to notify contacts (6 days after cases were likely infected). Jurisdiction 1, where our estimates of the absolute numbers of cases and hospitalizations averted by CICT was greatest (207 417-252 325 cases), CICT was responsible for just a 3.0% to 3.5% reduction in new infections per case (eTable 5 in Supplement 1).

We estimate that jurisdictions in the Midwest US averted the most cases on a per population basis because of CICT, averting between 1444 cases per 100 000 population (in our low-impact scenario) and 1600 cases per 100 000 (in our high-impact scenario) (Table 2). CICT programs among jurisdictions in the Western US averted the fewest number of cases and hospitalizations, between 488 cases per 100 000 (in our low-impact scenario) and 568 cases per 100 000 (in our high-impact scenario).

**Table 2.** CICT Performance and Estimated Outcomes, by US Census Region, November 25, 2020, to January 23, 2021^a^

US Census regions	No. of states^c^	Total population	Daily COVID-19 incidence per 100 000, mean (range)	Median (range)						
				CICT performance measure^b^			Estimated cases averted		Estimated cases averted per 100 000 population	
				Time from infection to isolation, d	Cases isolated, low, %	Cases isolated, high, %	Low	High	Low	High
Midwest	5	30 947 757	58 (42-75)	7 (6-7)	17 (15-25)	19 (18-28)	73 780 (19 577-121 865)	84 523 (23 221-158 766)	1444 (639-2213)	1600 (838-2727)
Northeast	5	25 348 752	59 (19-92)	7 (6-10)	16 (4-35)	19 (5-34)	32 084 (5921-66 362)	41 194 (7005-86 692)	900 (53-6139)	1155 (62-8183)
South	7	33 384 859	55 (22-88)	8 (7-12)	19 (14-41)	24 (16-49)	21 170 (5466-120 157)	27 473 (6452-156 557)	670 (80-1987)	895 (94-2590)
West	6	49 893 913	61 (28-94)	8 (7-9)	14 (4-23)	17 (5-24)	19 484 (4858-207 417)	24 326 (5721-252 325)	488 (271-704)	568 (336-856)
Total	23	139 575 281	58 (19-94)	7 (6-12)	17 (4-41)	19 (5-49)	22 014 (4858-207 417)	27 473 (5721-252 325)	704 (53-6139)	895 (62-8183)

Abbreviation: CICT, Case Investigation and Contact Tracing.

^a^ US Census regions are defined by the US Census Bureau.^11^

^b^ Days from infection to isolation were calculated using jurisdictions’ reported days from testing to case and contact notification, the COVID-19 incubation period, and assumptions regarding the timing of entry into isolation or quarantine after notification. Percentage of cases isolated was calculated from jurisdictions’ reported metrics on CICT program performance, such as the proportions of cases interviewed and contacts notified or monitored. The lower estimates assume a fraction of interviewed cases and contacts complied with isolation and quarantine guidelines, whereas the high estimates assume all interviewed cases and monitored contacts did so (Table 1 and eAppendix 1 in Supplement 1).

^c^ Includes 22 states (3 with a major city excluded) and 1 territory.

When we maximized compliance among interviewed cases and notified contacts (Table 1), we estimated that CICT could have averted 1.72 million cases and 42 2631 hospitalizations (approximately 26% greater than our high baseline estimate) across the 23 jurisdictions during the 60-day study period. Furthermore, when we accounted for the potential that some cases and contacts would have isolated and quarantined even without CICT program interviews or notification, we estimated that CICT would have averted 0.77 to 1.01 million cases and 18 998 to 24 845 hospitalizations (30% and 26% less than our baseline low-impact and high-impact estimates, respectively).

## Discussion

In this decision analytical model study, we estimated that CICT programs in 23 US jurisdictions potentially averted 1.11 to 1.36 million cases and 27 231 to 33 527 hospitalizations in a 60-day period during the 2020 to 2021 winter surge. There were 5 269 390 total cases reported across these jurisdictions during the same 60-day period, suggesting that CICT may have reduced the COVID-19 burden by 17% to 21%. Our range of estimates reflects uncertainties regarding the proportions of cases and contacts who effectively isolated or quarantined as a result of interactions with their health departments. Despite this uncertainty, our estimates of CICT performance were substantial, with averted cases exceeding 1 million across the 23 jurisdictions in our low-impact scenario.

Although our aggregate estimated count of averted cases and hospitalizations was sizeable, it was uneven across the jurisdictions. We estimated that CICT averted 1 of every 100 remaining cases not prevented by nascent vaccination efforts and other NPIs in the lowest performing jurisdiction and as many as 66 cases in the highest performing jurisdiction. We also found that population size was associated with our estimates of CICT performance. On average, our estimates suggest that jurisdictions with larger populations averted more cases, although this was expected given their larger populations eligible for protection. Conversely, the smallest jurisdictions were estimated to have averted the most cases on a per-population basis. This result may reflect, in part, that smaller jurisdictions were able to rely on existing CICT staff who had community knowledge and experience connecting with the population, whereas the caseloads in larger jurisdictions required hiring temporary, less experienced staff. A multivariable analysis, using data from several months of the pandemic, is needed to tease apart such factors. For example, population size alone cannot explain the variability in our estimated outcomes. Jurisdictions 5, 6, and 7 were in different population categories (with jurisdiction 5 being 10 times larger than jurisdiction 6), but all 3 jurisdictions were estimated to have averted approximately 87 000 cases under our high-impact scenario (Figure, panel A). Jurisdiction 6’s CICT program is also notable for averting the most cases per 100 000. This result reflects the jurisdiction’s success at interviewing cases (79% interviewed and >50% named at least 1 contact) and being among the fastest to notify contacts (6 days after cases were likely infected).

We also found regional differences in CICT performance. On the basis of the median averted case estimates per 100 000 population, Midwest jurisdictions’ CICT programs performed the best, whereas CICT programs in Western jurisdictions were least successful. Future studies exploring the potential reasons for these differences may consider incidence, factors affecting public acceptance of CICT (eg, sociodemographic makeup and cultural norms), and aspects of program implementation (eg, staffing levels and efficiency).

Our sizeable estimates of averted cases are partially due to the success of the analyzed CICT programs at suppressing the transmission not controlled by vaccination and other NPIs, compounded over approximately 10 generations of infection during our 60-day observation period. For example, in jurisdiction 1, where our estimates of the absolute number of cases and hospitalizations averted by CICT were greatest, CICT was estimated to be responsible for just a 3.0% to 3.5% reduction in new infections per case (eTable 5 in Supplement 1). However, jurisdiction 1 also had a very large burden of infectious cases at the start of our 60-day period and was one of the largest jurisdictions. This example shows that even when the percentage reduction in transmission from CICT is in the low single digits, when applied to large populations, the influence over multiple generations of cases is meaningful.

This analysis was possible because of the rich and unique programmatic data provided by jurisdictions and the use of assumptions to address key uncertainties, such as the compliance with public health recommendations. Still, important information was absent. As such, our results of CICT’s performance may overestimate or underestimate the true impact. Our estimates may be low because we do not account for the indirect effects of CICT programs on transmission reductions. For example, because of their interactions with health department staff, cases and contacts may have additionally notified and motivated isolation or quarantining among close contacts who, themselves, were not contacted by the CICT program. In addition, some individuals may have isolated or quarantined without being contacted, because of CICT program-funded ad campaigns or information obtained from their health department’s website. Other factors may have affected our estimates, although the direction of their impact is less clear. For example, we may have overestimated or underestimated CICT’s performance if the calculated number of contacts per case for each jurisdiction (eAppendix 1 in Supplement 1), the timing of testing and initiation of isolation or quarantine, or the compliance with public health recommendations differed from our examined scenarios.

This study was performed before the Delta or Omicron variants dominated transmission in the US and before vaccines were widely available. Increasing vaccination may be expected to reduce the absolute number of cases eligible to be averted by CICT. However, CICT’s performance (ie, percentage of cases isolated by CICT) would increase if jurisdictions were able to prioritize notification and monitoring of unvaccinated or undervaccinated populations, especially during periods of high caseloads. Furthermore, the performance of CICT can be potentially reduced when Delta or Omicron variants are predominantly circulating because of their shorter duration of infectiousness. Alternatively, the higher transmissibility of the Delta and Omicron variants potentially increases CICT’s performance since each averted case prevents more new infections than we originally allowed. The degree to which these factors offset one another is unclear.

### Strengths and Limitations

Our study has several strengths. First, the breadth of data on CICT implementation enabled us to generate a profile of CICT performance for nearly one-half of the US. By anonymizing jurisdictions and assessing the same time frame, we were able to present and compare the range of outcomes among 23 CICT programs spanning the country. Furthermore, this work can be replicated for other jurisdictions and time periods. The tool that we used, COVIDTracer Advanced,^9^ is provided (Supplement 2) and is designed for use by practicing public health officials. Jurisdictions can conduct site-specific analyses using these methods to estimate prevention impact, guide local public health programming, and reflect on resource utilization (eg, hospital beds).

Our study also has limitations. Jurisdictions’ self-reported CICT performance measures were not intended for this analysis. Although we used the previously described data quality checks (eFigure 2 in Supplement 1), the reported measures that we used were likely influenced by differences in jurisdictions’ surveillance systems, CICT platforms and protocols (eg, how they defined, enrolled, and monitored contacts). The extent to which these differences affected our results is unclear. We also only assess the CICT performance over 2 months (60 days) of the pandemic and in 23 US jurisdictions. Results may differ for other jurisdictions and periods (eg, during the Delta or Omicron surges and wider use of vaccine). Because cases were spiking across the entire US during the period that we analyzed and the vaccines had not yet been widely administered, it is likely that our estimates provide an upper limit of cases averted by CICT during the pandemic as of this writing (August 31, 2021). Also, because we used statewide data, our results dilute potentially meaningful differences in CICT performance within jurisdictions (eg, rural vs urban counties). In addition, the accuracy of our results may be affected by our model’s design. For example, the COVIDTracer Advanced model assumes homogeneous mixing among individuals in the population and does not account for any age-based or location-based heterogeneities in transmission (such as within and between households or schools), or variations in the effectiveness of vaccines and other NPIs over the study period. The extent of the influence of these factors, however, appears limited and would not appreciably alter our estimates or their implications for public health policy makers (eAppendix 5, eFigure 5, and eTable 6 in Supplement 1).

## Conclusions

Our analysis combined primary implementation data with mathematical modeling to estimate the health outcomes of COVID-19 CICT programs across nearly one-half of US states and territories. The volume of estimated cases and hospitalizations averted underscores the critical role CICT programs could play in curtailing the pandemic, whereas differences among jurisdictions illustrate the opportunities to further improve effectiveness. Case investigation and contact tracing remain CDC-recommended practices for personally communicating individualized prevention activities against COVID-19. This work quantifies for public health decision-makers the benefits from sustaining and improving these programs.

## Notes

Supplement 1. **eAppendix 1.** Case Investigation and Contact Tracing Effectiveness **eFigure 1.** Illustrative Example of the Timing of COVID-19 Case Isolation and Quarantine of Contacts **eTable 1.** Summary of Reported Case Investigation and Contact Tracing (CICT) Data Reported to CDC’s ELC Program and Calculated CICT Effectiveness for the 23 Jurisdictions Analyzed and All Funded Jurisdictions, 11/25/20 to 12/24/20 (30 Days) **eFigure 2.** Inclusion and Exclusion Criteria for Analysis of Jurisdictions **eAppendix 2.** COVIDTracer Advanced Model **eFigure 3.** COVIDTracer Advanced Model Structure **eTable 2.** Daily Percentage Risk of Transmission by Infectiousness State and Clinical Symptoms **eTable 3.** Epidemiological Parameters, Values, and Sources **eTable 4.** Assumed Proportion of Cases by Age Group and Infection-to-Hospitalization Rate, Default Values in COVIDTracer Advanced and Sources **eTable 5.** Estimated Impacts of Case Investigation and Contact Tracing (CICT) and Other Interventions From 11/25/20 to 1/23/21 (60 Days), by Jurisdiction and CICT Impact Scenarios **eAppendix 3.** Instructions for Using COVIDTracer Advanced Special Edition to Estimate the Number of COVID-19 Cases and Hospitalizations Averted by CICT **eAppendix 4.** Isolation/Quarantine Compliance Scenarios: Sources and Details **eFigure 4.** Estimated Hospitalizations Averted Due to CICT Programs From 11/25/20 to 1/23/21 (60 Days) **eAppendix 5.** Alternate Approaches to Simulating Epi-Curves Without CICT and Their Results **eFigure 5.** Fitted Epidemic Curve Outputs From COVIDTracer Advanced and Observed Data for the 60-Day Period, by Jurisdiction and Fitting Approach **eTable 6.** Effect of Alternate Fitting Methods on Estimates of the Impact of Case Investigation and Contact Tracing (CICT) from 11/25/20 to 1/23/21 (60 Days), Under “Low” CICT Impact Scenario
eReferences

Supplement 2.
COVIDTracer Advanced Special Edition
