# Supplementary material for: Estimated COVID-19 Cases and Hospitalizations Averted by Case Investigation and Contact Tracing in the US
Source: JAMA Netw Open. 2022 Mar 25;5(3):e224042. doi: 10.1001/jamanetworkopen.2022.4042 (PMC8956978; doi:10.1001/jamanetworkopen.2022.4042)
Supplement: Supplement 1. — eAppendix 1. Case Investigation and Contact Tracing Effectiveness eFigure 1. Illustrative Example of the Timing of COVID-19 Case Isolation and Quarantine of Contacts eTable 1. Summary of Reported Case Investigation and Contact Tracing (CICT) Data Reported to CDC’s ELC Program and Calculated CICT Effectiveness for the 23 Jurisdictions Analyzed and All Funded Jurisdictions, 11/25/20 to 12/24/20 (30 Days) eFigure 2. Inclusion and Exclusion Criteria for Analysis of Jurisdictions eAppendix 2. COVIDTracer Advanced Model eFigure 3. COVIDTracer Advanced Model Structure eTable 2. Daily Percentage Risk of Transmission by Infectiousness State and Clinical Symptoms eTable 3. Epidemiological Parameters, Values, and Sources eTable 4. Assumed Proportion of Cases by Age Group and Infection-to-Hospitalization Rate, Default Values in COVIDTracer Advanced and Sources eTable 5. Estimated Impacts of Case Investigation and Contact Tracing (CICT) and Other Interventions From 11/25/20 to 1/23/21 (60 Days), by Jurisdiction and CICT Impact Scenarios eAppendix 3. Instructions for Using COVIDTracer Advanced Special Edition to Estimate the Number of COVID-19 Cases and Hospitalizations Averted by CICT eAppendix 4. Isolation/Quarantine Compliance Scenarios: Sources and Details eFigure 4. Estimated Hospitalizations Averted Due to CICT Programs From 11/25/20 to 1/23/21 (60 Days) eAppendix 5. Alternate Approaches to Simulating Epi-Curves Without CICT and Their Results eFigure 5. Fitted Epidemic Curve Outputs From COVIDTracer Advanced and Observed Data for the 60-Day Period, by Jurisdiction and Fitting Approach eTable 6. Effect of Alternate Fitting Methods on Estimates of the Impact of Case Investigation and Contact Tracing (CICT) from 11/25/20 to 1/23/21 (60 Days), Under “Low” CICT Impact Scenario eReferences [file jamanetwopen-e224042-s001.pdf]

Supplemental Online Content

Rainisch G, Jeon S, Pappas D, et al. Estimated COVID-19 cases and hospitalizations averted by case investigation and contact tracing in the US. *JAMA Netw Open.* 2022;5(3):e224042. doi:10.1001/jamanetworkopen.2022.4042

eAppendix 1. Case Investigation and Contact Tracing Effectiveness
eFigure 1. Illustrative Example of the Timing of COVID-19 Case Isolation and Quarantine of Contacts
eTable 1. Summary of Reported Case Investigation and Contact Tracing (CICT) Data Reported to CDC's ELC Program and Calculated CICT Effectiveness for the 23 Jurisdictions Analyzed and All Funded Jurisdictions, 11/25/20 to 12/24/20 (30 Days)
eFigure 2. Inclusion and Exclusion Criteria for Analysis of Jurisdictions
eAppendix 2. COVIDTracer Advanced Model
eFigure 3. COVIDTracer Advanced Model Structure
eTable 2. Daily Percentage Risk of Transmission by Infectiousness State and Clinical Symptoms
eTable 3. Epidemiological Parameters, Values, and Sources
eTable 4. Assumed Proportion of Cases by Age Group and Infection-to-Hospitalization Rate, Default Values in COVIDTracer Advanced and Sources
eTable 5. Estimated Impacts of Case Investigation and Contact Tracing (CICT) and Other Interventions From 11/25/20 to 1/23/21 (60 Days), by Jurisdiction and CICT Impact Scenarios
eAppendix 3. Instructions for Using COVIDTracer Advanced Special Edition to Estimate the Number of COVID-19 Cases and Hospitalizations Averted by CICT
eAppendix 4. Isolation/Quarantine Compliance Scenarios: Sources and Details
eFigure 4. Estimated Hospitalizations Averted Due to CICT Programs From 11/25/20 to 1/23/21 (60 Days)
eAppendix 5. Alternate Approaches to Simulating Epi-Curves Without CICT and Their Results
eFigure 5. Fitted Epidemic Curve Outputs From COVIDTracer Advanced and Observed Data for the 60-Day Period, by Jurisdiction and Fitting Approach
eTable 6. Effect of Alternate Fitting Methods on Estimates of the Impact of Case Investigation and Contact Tracing (CICT) from 11/25/20 to 1/23/21 (60 Days), Under "Low" CICT Impact Scenario
eReferences

This supplemental material has been provided by the authors to give readers additional information about their work.

© 2022 Rainisch G et al. *JAMA Network Open*.

eAppendix 1. Case Investigation and Contact Tracing Effectiveness

The effectiveness of case investigation and contact tracing (CICT) is determined by the proportion of cases and their infected contacts that are effectively isolated and quarantined, preventing further transmission in the susceptible population. The duration of quarantine and isolation is described in Centers for Disease Control and Prevention (CDC)'s guidance.[9] We assumed that confirmed cases are effectively isolated following case interviews. We further assumed that contacts are quarantined upon either contact notification or through active monitoring.

We calculated the average proportion of cases and contacts isolated and quarantined by CICT for each location as follows:

Step 1: We first calculated the proportion of cases that effectively isolated:

$$Compliance * \left(\frac{\#\ Cases\ that\ completed\ case\ interview}{Total\ number\ of\ cases}\right) \qquad Term\ A$$

Step 2: We then calculated the proportion of infected contacts that effectively quarantined:

$$Compliance * \%\ Contacts\ identified * \%\ Contacts\ notified \qquad Term\ B$$

Where:

$$\%\ Contacts\ identified = \frac{\#\ Contacts\ named\ by\ interviewed\ cases}{Total\ number\ of\ contacts} \qquad Term\ B.1$$

and

$$\%\ Contacts\ notified = \frac{\#\ Contacts\ notified}{\#\ Contacts\ named\ by\ interviewe\quad cases} \qquad Term\ B.2$$

The "Total number of contacts" in *Term B.1* was the expected total number of contacts generated by all cases. We estimated it by multiplying the total cases reported by a jurisdiction by the average number of contacts per case as follows:

$$Total\ Cases * \left(\frac{Total\ \#\ Contacts\ named\ by\ interviewed\ cases}{\#\ Cases\ that\ named\ at\ least\ 1\ contact}\right) \qquad Term\ B.1.1$$

© 2022 Rainisch G et al. *JAMA Network Open*.

Step 3: We took the weighted average between the results of steps 1 and 2 (Terms A and B) by weighting quarantined contacts by R_0, since undetected infected contacts will infect R_0 additional individuals on average (or 2.5 new infections per infected contact). This resulted in the final equation:

Average proportion of cases and contacts (which become cases) isolated by CICT =
*[% Cases interviewed*Compliance + (R_0 * % Contacts identified * (% Contacts monitored *Compliance + % Contacts notified but not monitored*Compliance))] / (1+R_0)*

By populating this equation with the assumed compliance to isolation/quarantine guidance (described in Table 1), we assessed the following three scenarios.

Equation 1: Baseline Low Estimate

80% of interviewed cases and monitored contacts, and 30% of notified contacts (who are not monitored), isolate or quarantine:

Average proportion of cases and contacts (which become cases) isolated =
[% Cases interviewed*0.8 + (R_0 * % Contacts identified * (% Contacts monitored *0.8 + % Contacts notified but not monitored*0.3))] / (1+R_0)

Equation 2: Baseline High Estimate

100% of interviewed cases and monitored contacts isolate or quarantine:

Average proportion of cases and contacts (which become cases) isolated =
[% Cases interviewed + (R_0 * % Contacts identified * % Contacts monitored)] / (1+R_0)

Equation 3: Sensitivity Analysis (Maximum CICT Impact) Estimate

100% of interviewed cases and 100% of contacts isolate or quarantine:

Average proportion of cases and contacts (which become cases) isolated =
[% Cases interviewed + (R_0 * % Contacts identified * % Contacts notified)] / (1+R_0)

where R_0 is the assumed number of new infections per case without any interventions and when the population is entirely susceptible to infection (Table A2).

© 2022 Rainisch G et al. *JAMA Network Open*.

In addition, reducing the time from case identification to effective isolation is critical for case investigation and contact tracing to succeed. The longer the cases and contacts interact with the susceptible population, the greater the opportunity for onward transmission. In practice, cases with no known exposure are predominantly identified and isolated after symptom onset[1], and cases with known exposures (*i.e.,* contacts that eventually become infected cases) can begin quarantine upon contact notification (even potentially prior to symptom onset). We assumed asymptomatic cases can only be identified and isolated if they are notified through case investigation and contact tracing. For the purposes of our study, we assumed the proportions of cases with no known exposure and cases with known exposures were equal (*i.e.*, 50/50 breakdown) because we did not have data on what prompted case identification in each location. Therefore, for each location the days to effective case isolation was determined by taking the average of the days to effective isolation between case groups with known and no known exposures. The time to effective case isolation for each of the two case groups was determined as follows:

For symptomatic cases with no known exposures (*i.e.,* symptoms prompt identification):

We assumed that cases experience a 5-day pre-symptomatic period (See Table A2), get tested the day after symptom onset (*i.e.,* 6 days would have transpired since infection at the time of testing). We then obtained the number of days from testing to result notification by adding the reported "*Median days from specimen collection to case reporting to the health department (HD)"*. We also assumed that confirmed cases begin isolation the day after their result notification (*i.e.,* we added 1 to the total obtained above). Our assumptions regarding the "next-day" timing of testing and entry into isolation are based on symptoms and notifications beginning or occurring throughout the day, with a sizeable portion occurring sufficiently late enough in the day to prevent testing and entry into isolation the same evening. This assumption takes into account practical considerations such as time needed to find a testing site and arrange an appointment, and for notified individuals to prepare to isolate (*e.g.,*

---

[1] Some cases can be identified before being symptomatic (*e.g.,* during screening for various reasons)

© 2022 Rainisch G et al. *JAMA Network Open*.

purchasing food or medications, setting up childcare, handling work or other commitments).

For cases with known exposures (*i.e.,* those who were notified they were a contact and eventually became a case):

We first calculated the days from index case testing to their exposed contacts' notification by summing jurisdictions' reported "*Median days from specimen collection to case report to the HD*", "*Median days from case report to the HD to the case interview completion*", and "*Median days from case interview completion to contacts notification*". We assumed that contacts begin quarantine the day after receiving exposure notification from their health department (*i.e.,* we added 1 to the sum above). The "next-day" timing of entry into quarantine is based on the same practical reasoning as cases needing time to prepare to isolate once notified (described above). We then used the resultant sum from the procedure above to estimate the time (in days) from exposure to quarantine for contacts. Because we did not have information on when exposures actually occurred for contacts, we assumed that these individuals' exposures occurred at the midpoint of their potential exposure window (in days). We identified the earliest date in this window as the first day of infectiousness among cases to which contacts were exposed. Based on our assumed 5-day pre-symptomatic period for symptomatic cases (described above), this was two days prior to the symptom onset date in cases exposing the contact. We identified the latest possible exposure as the date the cases exposing them were interviewed by the health department (because they began isolation the next day). See both "Contacts" rows in Figure A2 for a visual depiction of this timeline.

© 2022 Rainisch G et al. *JAMA Network Open*.

eFigure 1. Illustrative Example of the Timing of COVID-19 Case Isolation and Quarantine of Contacts

	Day 1	2	3	4	5	6	7	8	Day 9	Day 10		Days from Exposure to Isolation
**Index Case**	Exposed			Contagious Period Begins		Symptom Onset	Tested	Result Notification & Case Interview	Begin Isolation			8
**Contacts** (Earliest possible exposure)				Exposed					Exposure Notification	Begin Quarantine		6
**Contacts** (Latest possible exposure)								Exposed	Exposure Notification	Begin Quarantine		2

Notes: In this hypothetical scenario, we assume a jurisdiction needed 1 day from specimen collection (testing) to result notification and 2 days from specimen collection to contact notification. The index case (symptomatic case with no known exposure) began showing symptoms on day 6 post-infection, got tested on day 7 and was notified of test result on day 8. The case's contacts (cases with known exposure) were exposed sometime between days 4 to 8 and notified of their exposure on day 9. Therefore, the index case began isolation on day 9 and contacts went into quarantine on day 10 (based on our assumptions above). To calculate the days from contacts' exposure to their quarantine, we took the average of the maximum days a contact was infected (6 days in this example based on the earliest possible exposure) and the fewest days the contact could be infected (2 days in this example, based on the latest possible exposure), and weighted each day span by the case's infectiousness on each of possible exposure days. The result is 3.9 days in this example, meaning the contact had been exposed for 3.9 days upon initiating quarantine. We then took the average between 8 days (index case) and 3.9 days (contacts) as the number of days from exposure to isolation (for both cases and contacts). This is 6 days in this example.

The days between cases with known exposures becoming infected and their exposure notification can vary from what we assumed. For example, cases may take longer to become symptomatic, or get tested the same day that they become symptomatic or begin their isolation on the same day as their results notification. Similarly, contacts who become cases may be exposed earlier or later than we assumed and may make up a larger or smaller share of the case pool. Readers interested in more detail of the influence of varying our assumed time to case isolation may wish to see Table A6 in the Technical Supplement of our 14-site study on CICT impact, containing results of a sensitivity analysis examining this topic in those jurisdictions.[10]

© 2022 Rainisch G et al. *JAMA Network Open*.

CDC’s Epidemiology and Laboratory Capacity (ELC)-funded jurisdictions also reported the *Number of contacts that were notified within 1 day of case interview*, the *Number of contacts that were notified between 1-3 days after case interview*, and the *Number of contacts that were notified within 3 or more days after case interview*. We used these additional data elements as a quality check (Figure A3) of the reliability of jurisdictions’ reported median values regarding notification timing (described above). We did this by calculating the lower limit of the average number of days from case interview to contact notification as follows:

$$
\begin{aligned}
&0.5\ days * (\%\ contacts\ notified\ within\ 1\ day) \\
&+ 2\ days * (\%\ contacts\ notified\ between\ 1 - 3\ days) \\
&+ 3\ days * (\%\ contacts\ notified\ 3\ or\ more\ days\ after\ case\ interview).
\end{aligned}
$$

This metric assumes that all contacts were notified within 3 days of the case interview. We used this metric to exclude jurisdictions from the analysis (*i.e.,* deemed reported data unreliable) when the lower limit of the average time to contact notification was greater than our calculated time to contact notification using reported median days AND the proportion of contacts that were notified 3 or more days after case interview was less than 10% of total contacts (*i.e.,* too few to exert enough influence on the average lower limit for it to plausibly exceed the median-based value).

© 2022 Rainisch G et al. *JAMA Network Open*.

eTable 1. Summary of Reported Case Investigation and Contact Tracing (CICT) Data Reported to CDC's ELC Program and Calculated CICT Effectiveness for the 23 Jurisdictions Analyzed and All Funded Jurisdictions, 11/25/20 to 12/24/20 (30 Days)

Measures	Median (Interquartile Range)	
	23 Jurisdictions analyzed	All Jurisdictions[a]
**Reported CICT ELC Program Data**		
% of cases interviewed	49% (39 – 67%)	58% (39 – 74%)
% of interviewed cases who named their contacts	25% (15 – 35%)	27% (15 – 47%)
% of contacts who were notified	59% (37 – 72%)	64% (35 – 84%)
% of contacts who were monitored	32% (17 – 50%)	48% (29 – 78%)
Reported days from testing to case interview	3.5 days (3.0 – 5.0)	3.0 days (2.4 – 5.0)
Reported days from testing to contact notification	4.0 days (3.0 – 5.2)	4.0 days (3.0 – 5.7)
**Calculated CICT Effectiveness**		
% of cases and contacts isolated/quarantined (high)	19% (16 – 25%)	N/A
% of cases and contacts isolated/quarantined (low)	17% (14 – 22%)	
Calculated days from exposure to isolation/quarantine	7.0 days (7.0 – 8.0 days)	

[a] Out of 64 total ELC jurisdictions, 5 did not report CICT program data and 3 reported a zero COVID-19 case count. Summary metrics are based on the remaining 56 ELC jurisdictions that reported the following measures: % of cases interviewed (*n=54*); % of interviewed cases who named their contacts (*n=52*); % of contacts that are notified (*n=53*); % of contacts that are monitored (*n=43*); Reported days from testing to case interview (*n=48*); Reported days from testing to contact notification (*n=45*).

© 2022 Rainisch G et al. *JAMA Network Open*.

eFigure 2. Inclusion and Exclusion Criteria for Analysis of Jurisdictions

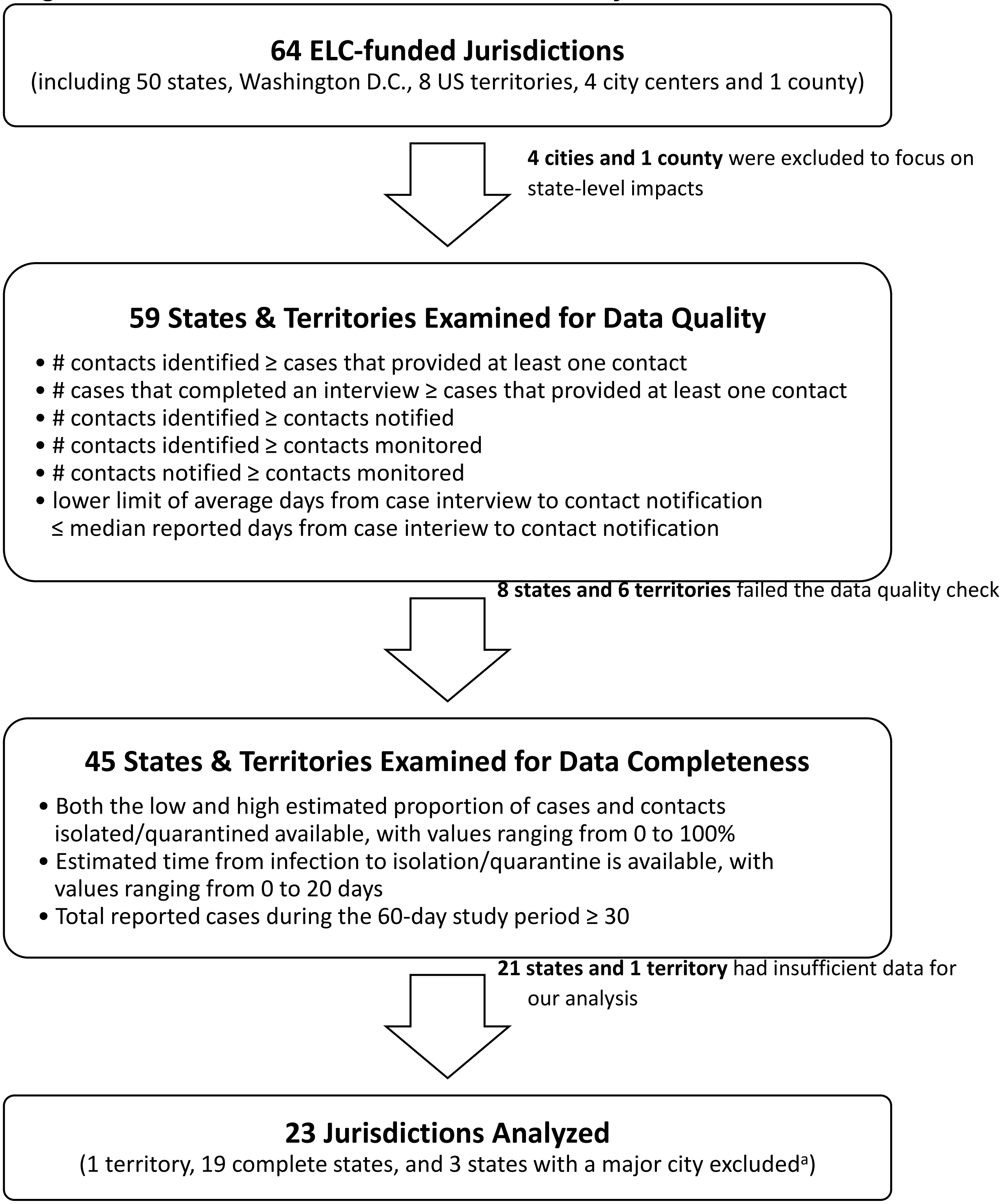

[a] Three states included a major city or county that were separately funded by the CDC's ELC program. Their reported CICT metrics are exclusive of the separately-funded locals.

© 2022 Rainisch G et al. *JAMA Network Open*.

eAppendix 2. COVIDTracer Advanced Model

COVIDTracer Advanced[1] is a spreadsheet-based compartmental Susceptible-Exposed-Infectious-Recovered (SEIR) epidemiological model, which illustrates the spread of a pathogen, resultant disease, and impact of interventions in a user-defined population. Readers can download the tool and enter input values of their choosing, exploring the impact of scenarios and assumptions beyond those covered in this manuscript. To model the clinical progression and transmission of disease using COVIDTracer Advanced, we used the following definitions and assumptions. A "case" was defined as a person who has been exposed, infected and subsequently becomes infectious, regardless of the presence of clinical symptoms. We assumed that for the first 3 days after infection, cases do not infect others. During days 4–5 post-infection, cases are pre-symptomatic but shed virus in amounts that may infect others.[2-5] During days 6–14, the infected person can be symptomatic and shedding virus, albeit during days 11–14 the risk of onward transmission is relatively low but non-zero (the complete infectivity distribution is given in Table A1). We assumed that approximately 40% of cases are asymptomatic during days 6-14 yet have a risk of onward transmission equal to 75% of symptomatic cases (Table A2) without vaccine or other non-pharmaceutical interventions (NPIs).[5] The model assumes homogeneous mixing among individuals and does not account for any age- or location-based heterogeneities in transmission.

© 2022 Rainisch G et al. *JAMA Network Open*.

eFigure 3. COVIDTracer Advanced Model Structure

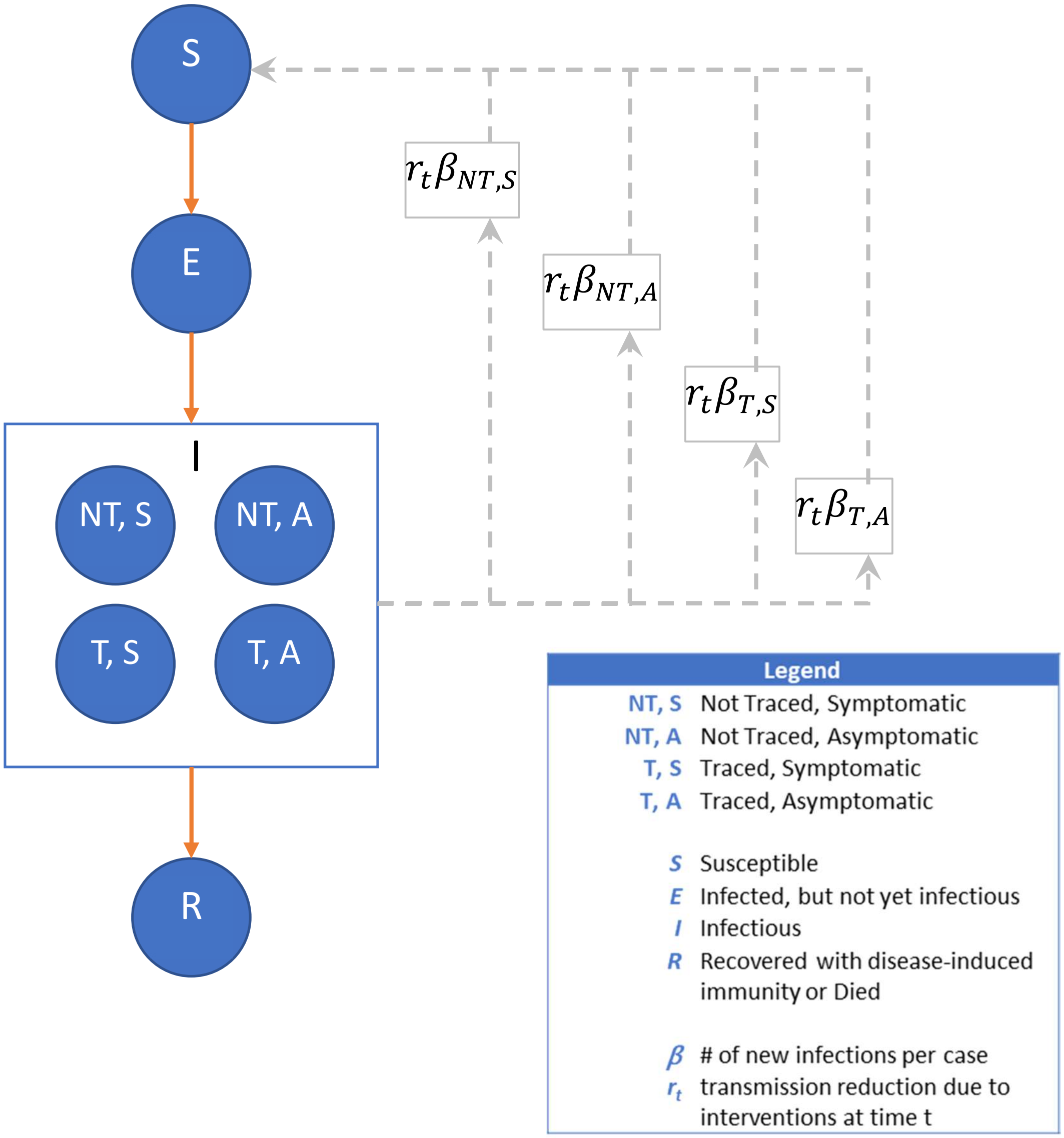

Notes: The model consists of individuals who are either *Susceptible* (S), *Infected but not yet Infectious* (E), *Infectious* (I), *Recovered or Died* (R). Individuals can move between these compartments as indicated by the orange arrows. The model tracks the number of individuals moving between these categories every day of the outbreak. The rate of new infections is influenced by the number of individuals in the *Infectious* (I) category (depicted by the light grey dashed lines). There are 4 types of *Infectious* individuals: cases (symptomatic or asymptomatic) who adhere to isolation guidelines because they were engaged by their health departments via case investigation and contact tracing efforts (CICT), and cases (symptomatic or asymptomatic) who do not participate in CICT efforts. The overall risk to the *Susceptible* population of onward transmission is dependent upon both the distribution of cases among these 4 infectious categories on each day, and any reductions in transmission associated with a jurisdiction's implementation of CICT, and vaccine and other non-pharmaceutical interventions.

© 2022 Rainisch G et al. *JAMA Network Open*.

eTable 2. Daily Percentage Risk of Transmission by Infectiousness State and Clinical Symptoms

Days post infection	Daily percentage risk of onward transmission[a] (%)	Infected person's state
1	0.00	*Infected, not yet infectious*
2	0.00	
3	0.00	
4	16.78	*Infectious, pre-symptomatic*
5	18.03	
6	17.07	*Infectious, symptomatic*
7	14.52	
8	11.27	
9	8.10	
10	5.48	
11	3.55	
12	2.26	
13	1.46	
14	1.48	
**Total**	**100**	

[a] Percentages show when onward transmission might occur by day of infectiousness
Sources: He *et al.*[2, 3] and Ferretti *et al.*[4] See also COVIDTracer Advanced manual.[1]

eTable 3. Epidemiological Parameters, Values, and Sources

Parameter	Default Value	Source
Infected but not yet infectious period	3 days	CDC COVID-19 Pandemic Planning Scenarios[5]
Pre-symptomatic and contagious (infectious) period	2 days	He *et al.*[2, 3], Ferretti *et al.*[4]
Symptomatic and contagious (infectious) period	9 days	He *et al.*[2, 3], Ferretti *et al.*[4]
New infections per case (R_0)	2.5	CDC COVID-19 Pandemic Planning Scenarios[5]
% of cases that are asymptomatic	40%	CDC COVID-19 Pandemic Planning Scenarios[5]
Infectiousness of asymptomatic cases (relative to symptomatic cases)	75%	CDC COVID-19 Pandemic Planning Scenarios[5]

eTable 4. Assumed[a] Proportion of Cases by Age Group and Infection-to-Hospitalization Rate, Default Values in COVIDTracer Advanced and Sources

Age group (year)	% of Total Cases	Source	% of all cases admitted to hospital care	Source
0 to 17	15	CDC COVID Data Tracker[6]	0.21	CDC COVID-19 Response Team[7], Wu *et al.*[8]
18 to 64	55		2.17	
65+	30		4.12	

[a] derived September 2020 using sources available at that time

© 2022 Rainisch G et al. *JAMA Network Open*.

eTable 5. Estimated Impacts of Case Investigation and Contact Tracing (CICT) and Other Interventions From 11/25/20 to 1/23/21 (60 Days), by Jurisdiction and CICT Impact Scenarios

Jurisdiction	Low CICT impact[b]					High CICT impact[b]				
	% Transmission reduction from		Cases Averted by CICT[e], 60 days	Hospitalizations Averted by CICT[e], 60 days	% Reduction in cases and hospitalizations by CICT[f], 60 days	% Transmission reduction from		Cases Averted by CICT[e], 60 days	Hospitalizations Averted by CICT[e], 60 days	% Reduction in cases and hospitalizations by CICT[f], 60 days
	Other NPIs & Vaccine[c]	CICT[d]				Vaccine & Other NPIs[c]	CICT[d]			
**1[a]**	53.6	3.0	207,417	5,097	12.8	53.3	3.5	252,325	6,200	15.1
**2**	53.6	8.7	121,865	2,995	37.5	52.6	10.5	158,766	3,901	43.9
**3**	51.6	9.8	120,157	2,953	42.6	50.5	11.8	156,557	3,847	49.1
**4**	56.5	5.7	97,231	2,389	23.8	56.3	6.2	107,689	2,646	25.7
**5**	54.1	3.5	70,297	1,727	15.8	53.8	4.3	90,217	2,217	19.4
**6**	49.4	13.6	65,037	1,598	51.6	47.8	16.2	86,692	2,130	58.7
**7[a]**	59.7	5.6	73,780	1,813	22.2	59.4	6.3	84,523	2,077	24.6
**8**	61.0	5.0	63,813	1,568	20.2	60.4	6.3	83,647	2,055	24.9
**9**	54.7	3.6	66,362	1,631	16.9	54.4	4.3	80,059	1,967	19.7
**10**	54.2	4.0	32,084	788	18.9	53.8	5.0	41,194	1,012	23.0
**11**	55.5	7.1	21,170	520	32.0	54.7	8.9	28,595	703	38.8
**12**	50.1	2.0	24,011	590	10.0	49.9	2.2	27,473	675	11.3
**13**	59.2	5.7	22,014	541	23.7	58.9	6.4	25,359	623	26.4
**14**	53.2	0.9	19,691	484	4.4	53.1	1.2	24,455	601	5.5
**15**	61.3	2.3	19,277	474	9.6	61.1	2.9	24,197	595	11.8
**16**	53.6	8.3	19,577	481	36.0	53.0	9.4	23,221	571	40.1
**17**	50.1	9.2	11,135	274	41.1	49.1	11.0	14,586	358	47.8
**18**	59.8	3.3	13,248	326	13.9	59.8	3.5	14,102	347	14.6
**19**	54.2	4.4	10,200	251	19.1	53.7	5.4	13,247	326	23.5
**20**	49.3	17.0	13,560	333	65.8	51.7	12.9	8,304	204	54.1
**21[a]**	60.4	0.4	5,921	145	1.3	60.4	0.4	7,005	172	1.6
**22**	58.6	0.4	5,466	134	1.5	58.6	0.5	6,452	159	1.7
**23**	62.9	3.4	4,858	119	13.1	62.7	3.9	5,721	141	15.1

[a] Single large city or county in these states were separate Epidemiology and Laboratory Capacity (ELC) jurisdictions and not included in this analysis.
[b] Low CICT impact scenario assumes only actively monitored contacts (who later became cases) effectively quarantined/isolated. High CICT impact scenario assumes notification prompted contacts (who later became cases) to quarantine effectively. In both scenarios we assumed interviewed cases fully adhered to isolation guidelines.
[c] Percent reduction in the number of new infections per case (R_t) due to a combination of vaccination and all other nonpharmaceutical interventions (NPIs; *e.g.,* masks use, social distancing, school/restaurant closures, etc. Calculated as the percent difference in R_0 and R_t after implementation of vaccine and other NPIs.
[d] Percent reduction in the number of new infections per case (R_t) due to CICT after the implementation of other NPIs. Calculated as the percent difference between R_t after implementation of other NPIs and R_t after implementation of both other NPIs and CICT.
[e] After accounting for the impacts from vaccination and all other NPIs.
[f] Cases or hospitalizations averted by CICT out of the estimated cases or hospitalizations remaining after the implementation of vaccination and other NPIs.

© 2022 Rainisch G et al. *JAMA Network Open*.

eAppendix 3. Instructions for Using COVIDTracer Advanced Special Edition to Estimate the Number of COVID-19 Cases and Hospitalizations Averted by CICT

These instructions will guide a user how to use the Special Edition version of COVIDTracer Advanced tool to repeat the analysis described in this manuscript to estimate COVID-19 cases averted by case investigation and contact tracing activities. The Special Edition version of COVIDTracer Advanced is a modification of the publicly available tool on CDC's website that enables users to assess the impact of CICT before vaccine was widely available. Additional modifications would be required if you intend to explicitly account for vaccinated individuals (*e.g.,* decreasing susceptible population over time, decreased risk of hospitalization among vaccinated individuals, etc).

Readers seeking basic information about the model, data elements, and definitions should refer the COVIDTracer Advanced User Manual. However, some statements in the web manual are not applicable to the Special Edition version used in this analysis. https://www.cdc.gov/coronavirus/2019-ncov/php/contact-tracing/COVIDTracerTools.html

COVIDTracer Advanced uses the Windows operating system (Microsoft Windows 2010 or higher) and Excel (Microsoft Office 2013 or higher).

Before starting, complete the following:

1) Determine your 60-day study period. The first day of your study period is your "model start date." This "model start date" will be referenced later in these instructions. For example, if you are interested in estimating cases and hospitalizations averted by CICT during the 60-day period from January 1-March 1, 2021, your "model start date" is January 1, 2021.

2) Obtain these data for the jurisdiction of interest:
    a. Total population
    b. Total cases as of the day before the model start date (In the example study period above, this is the total cases reported as of December 31, 2020.)
    c. Cases reported during the past 14 days (In the example study period above, this is the sum of cases reported from December 18 to 31, 2020.)
    d. The case trend during the past 14 days (*e.g.,* increasing, plateaued, decreasing)
    e. Daily (*i.e.,* incident) case counts for the 60-day study period
    f. The following case investigation and contact tracing program metrics. These metrics are meant to be representative of the 60-day study period. If you don't have such data for the entire study period, you may base these metrics on a shorter period (*e.g.,* 30 days or 4-weeks) from the model start date (and assume they are representative of the full 60 days):

© 2022 Rainisch G et al. *JAMA Network Open*.

i. Number of days from exposure to case isolation and contact quarantine
ii. Percent (%) of all cases successfully isolated and contacts quarantined

3) Open the COVIDTracer Advanced_SpecialEdition tool (Supplement 2)
   a. When opening the spreadsheet file, click the “Enable Macros” button for full functionality of the tool.
   b. Enable Excel “Solver Add-In.” **Instructions**: in Excel, click on File → Options → Add-ins → select “Analysis ToolPak” → click “Go” (not the “Ok” button) → select checkbox for “Solver Add-In” and click “Ok.”

   The Solver button, ?→ Solver will appear in the “Data” menu.

In worksheet, “A. Outbreak Details”

Step 1: Enter the population for the jurisdiction of interest.

Enter the population of your jurisdiction

Total Population	1,000,000	*persons*

Step 2: Enter the model start date, the total number of COVID-19 cases in the jurisdiction until the day before the model start date, and the number of cases reported in the last 14 days within the jurisdiction.

Enter information about case counts in your jurisdiction

Start Date	1/1/2021	
Total Cases as of 12/31/2020	35,000	*cases*
Cases in the last 14 days (from 12/18/2020 to 12/31/2020)	5,000	*cases*

Note: These data inputs will only create curves for the purpose of calculating resources needs. They are not intended as, nor should be intepreted as, forecasts of future cases

Step 3: Set the pattern of daily cases over the past 14-day period selected in Step 3.

The default is “Daily case counts are slowly increasing.” However, if daily case counts have been changing rapidly, remaining constant, or decreasing over the last 14 days, select from the pull-down menu the pattern that best matches the jurisdiction’s data.

The selection of the case trend in the past 14 days determines how reported cases are distributed over the 14 days prior to the model’s initiation date. Visually inspect the case trend and choose the most appropriate option. You can also run the model with different case trend patterns and pick one that yields the “best fit” (by repeating steps 3 to 6).

© 2022 Rainisch G et al. *JAMA Network Open*.

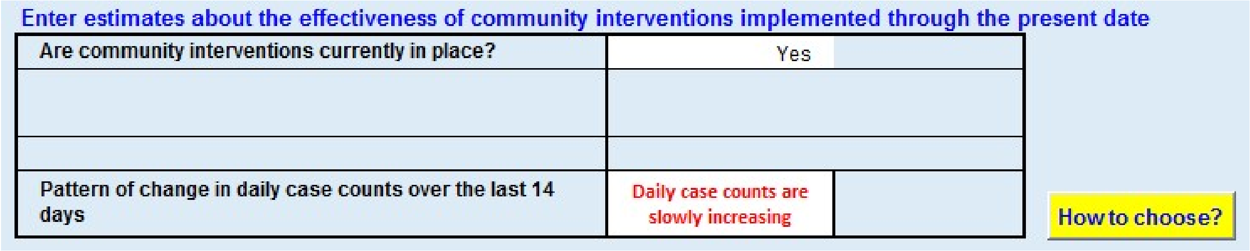

In worksheet, “Case Counts”

Step 4: Paste the jurisdiction’s daily case counts (*i.e.,* incident cases) for the 60-day study period into the “Daily” column (column AH)

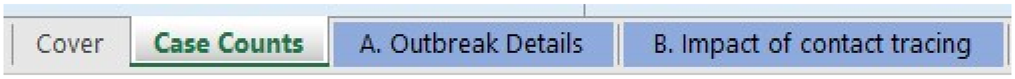

In Worksheet, “B. Impact of Contact Tracing”

Step 5: Using your representative CICT program data, enter values for:

- Number of days after infection that case is isolated
- % of all cases successfully isolated and contacts traced and monitored

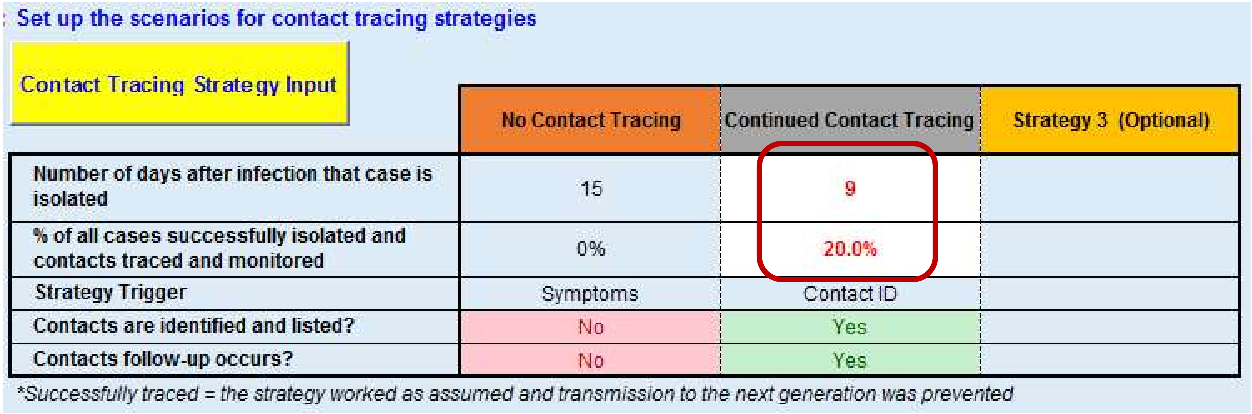

Step 6: Estimate the % reduction in transmission due to community interventions (shown in cell G28) by fitting COVIDTracer Advanced’s simulated curve to your observed case curve. You will use the Solver Add-in to do this: The Solver Add-in finds an optimal solution for the % reduction in transmission due to community intervention by minimizing the mean squared error (a mathematical value describing the differences between both curves; shown in cell O32).

Instructions for using the Solver:

© 2022 Rainisch G et al. *JAMA Network Open*.

From the Excel menu tab, click “Data” and the “Solver” button, then follow the instructions described here to set up the parameters in the pop-up dialogue box (see screen shot below):

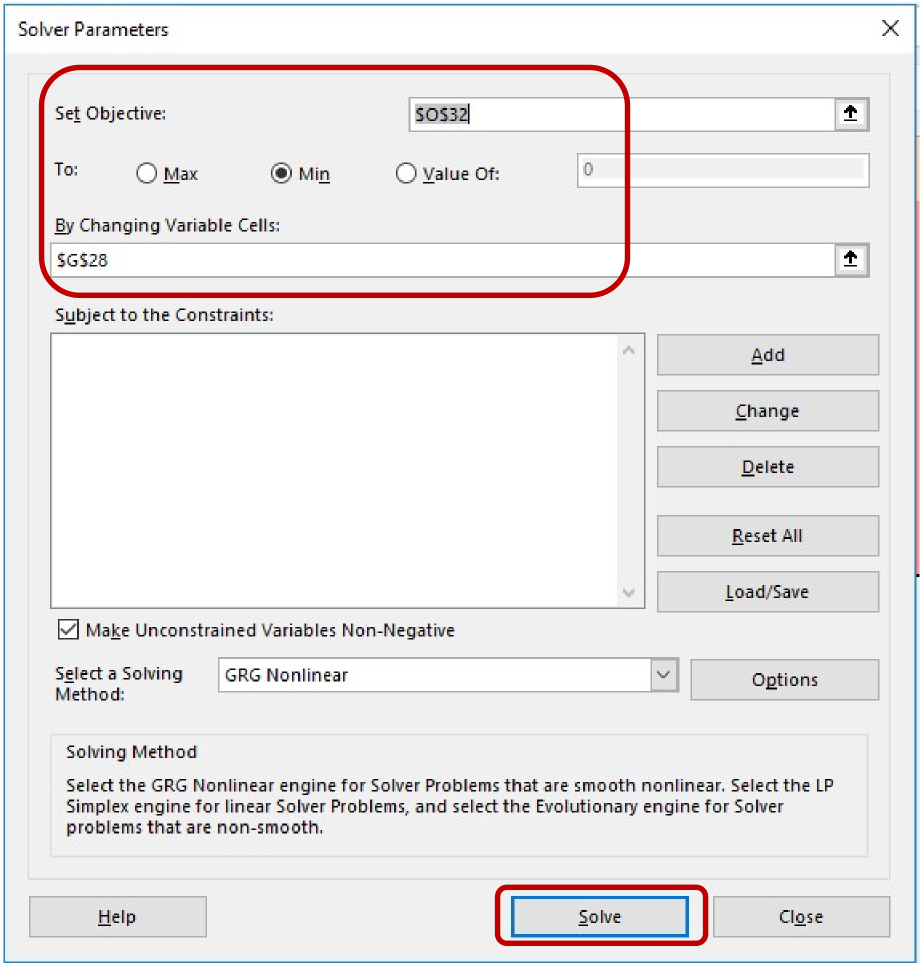

Set Objective: Set objective to cell “O32”, which is the mean squared error.

To: Select “Min”.

By Changing Variable Cells: Enter G28 (This cell refers the Solver to the “Estimated % reduction in transmission due to continued community interventions.”)

Select a Solving Method: For simplicity, we recommend selecting “GRG Nonlinear” from the drop-down menu.

Click “Solve” button.

Then the Excel Solver function will automatically find the optimal value (estimated % reduction in transmission due to continued community intervention) and populate the value in cell G28. The figure below shows a fitted curve (solid line) generated by

© 2022 Rainisch G et al. *JAMA Network Open*.

COVIDTracer Advanced after Step 6, that minimizes deviation from the reported case counts (dashed line).

Example Figure: Fitted curve using COVIDTracer Advanced

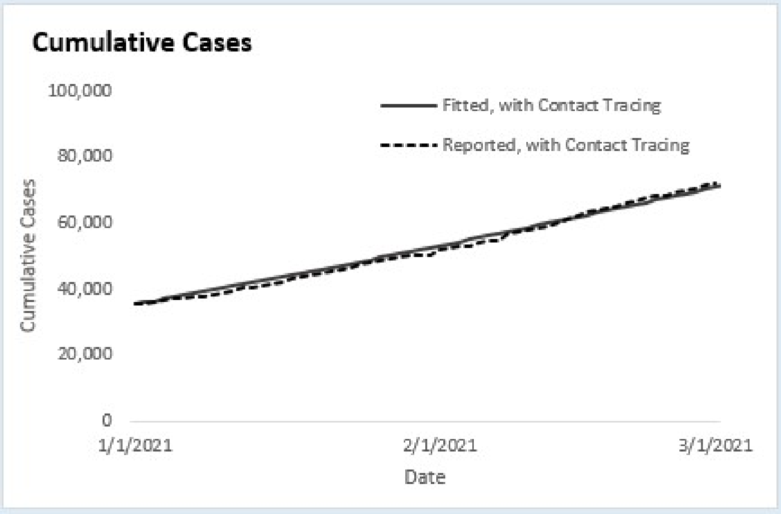

In Worksheet, "Results – Cases Averted"

Step 7. Users can find the % reduction in transmission due to CICT, and those that are attributable to all other interventions. The estimated number of cases and hospitalizations averted by CICT are also provided on this page.

Transmission Fraction

Transmission Reduction from Contact Tracing	4.5%
Transmission Reduction from All Other Interventions	54.7%
Remaining Transmission*	43.3%

*Calculated as follows: (1-reduction from CT) * (1-reduction from other interventions)*

Cases Averted, 60 days

Cases Averted by Contact Tracing	8,937
Cases Averted per 100,000 population	894
% of Additional Cases Averted by Contact Tracing**	19.7%

** *Additional cases averted by contact tracing out of every 100 remaining cases after accounting for the impact of all other interventions (e.g., vaccination, facemask policies, social distancing).*

Hospitalizations Averted, 60 days

Hospitalizations Averted by Contact Tracing	220
Hospitalizations averted per 100,000 population	22
% of Additional Hospitalizations Averted by Contact Tracing***	19.7%

*** *Additional hospitalizations averted by contact tracing out of every 100 remaining hospitalizations after accounting for the impact of all other interventions.*

© 2022 Rainisch G et al. *JAMA Network Open*.

eAppendix 4. Isolation/Quarantine Compliance Scenarios: Sources and Details

A review of multiple cross-sectional population surveys in the UK suggests 40-45% of people who had COVID-like symptoms self-reported fully complying with isolation guidance during their infectious periods.[11] Another survey in the US found that 85% of respondents who had COVID-like symptoms or tested positive stayed home (according to CDC guidelines) except to get medical care.[12] And a third survey, also in the US, found that 93% of adults said they would definitely (73%) or probably (20%) quarantine themselves for at least 14 days if told to do so by a public health official because they had the coronavirus (*i.e.,* they were confirmed cases; not just exposed contacts).[13]

© 2022 Rainisch G et al. *JAMA Network Open*.

Figure 4. Estimated Hospitalizations Averted Due to CICT Programs From 11/25/20 to 1/23/21 (60 Days)

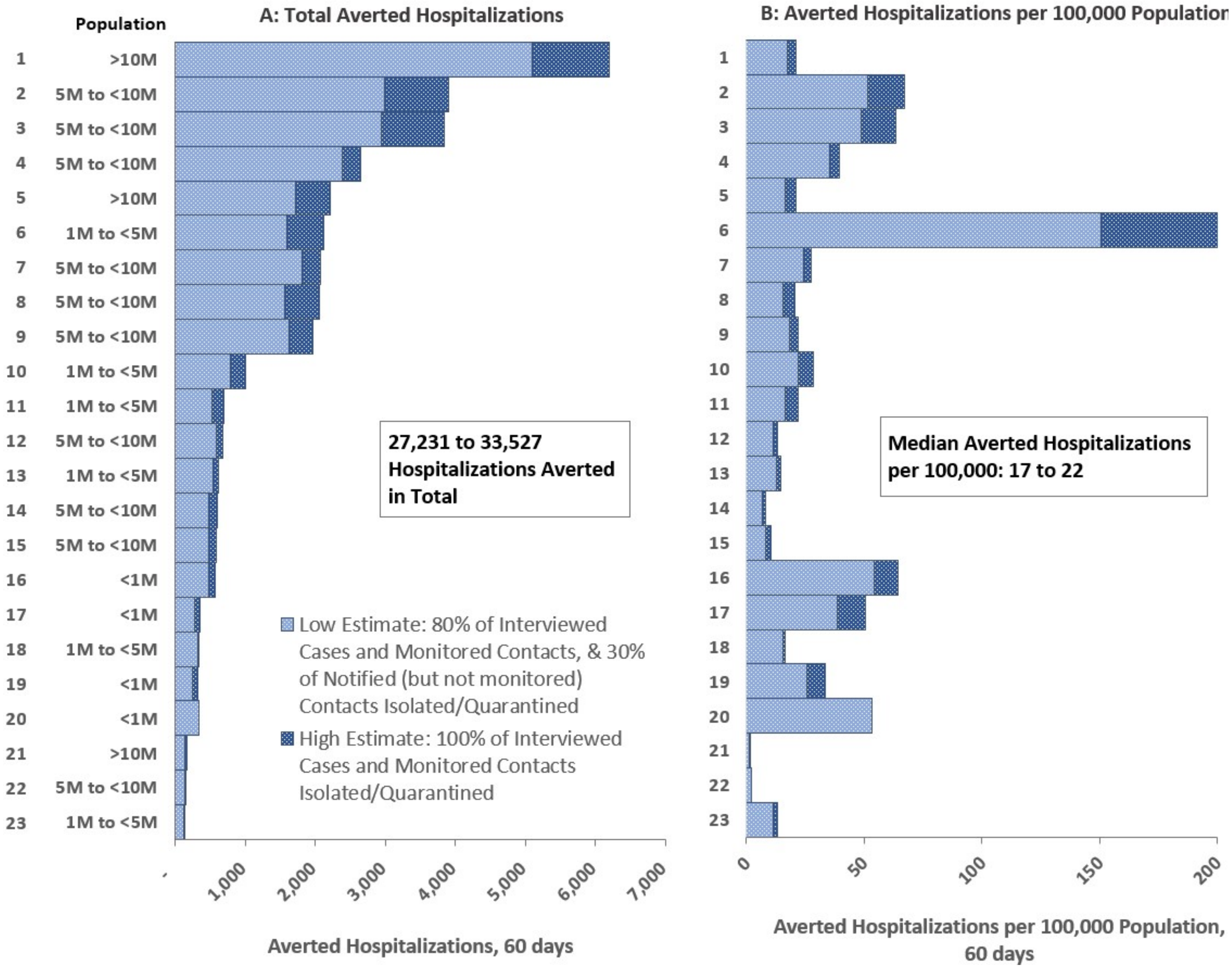

© 2022 Rainisch G et al. *JAMA Network Open*.

eAppendix 5. Alternate Approaches to Simulating Epi-Curves Without CICT and Their Results

We estimated the combined effectiveness of vaccine and other non-pharmaceutical interventions (NPIs) by fitting our model to cumulative cases and assuming that the effectiveness of NPIs remained constant over the course of our 60-day evaluation period. These choices enabled us to 1) avoid the influence of transient testing accessibility and test-seeking behaviors, or data reporting artifacts (observed in many locations around the Christmas and New Year's holidays), and 2) maintain COVIDTracer Advanced's accessibility and ease-of-use for practicing public health officials. However, fitting to cumulative cases weights early cases over later cases and can inflate model fit. Also, fixing the effectiveness of NPIs may result in an over- or under-estimation of impact. We, therefore, conducted an excursion analysis to examine the influence of these choices on our estimates of averted cases and hospitalizations.

We selected eight jurisdictions for this analysis: the five with the largest estimates of averted cases (accounting for 56% of total averted cases), and three others exhibiting clear and large changes in the overall trend of incident cases within our 60-day evaluation period (jurisdictions 1-5, 7, 12, and 20 in eTable 5).

For these eight jurisdictions, we repeated our fitting process, but used the incidence epi-curves, and fit up to three periods using our low CICT impact scenario (Table 1). The number of periods and their lengths (in days) were determined by visually examining and selecting inflection points in the 7-day moving average of the observed incidence curves of reported cases (eFigure 5).

This fitting procedure reduced discrepancies between the observed cumulative case count and the fitted curve's count on the last day of our evaluation period (which we use for calculating CICT impact on cases) for 6 of the 8 evaluated jurisdictions (eFigure 5). Based on the new fits, our averted case estimates decreased for five of the eight states and increased for the remaining three (eTable 6). Across all eight states, we estimate that CICT potentially averted 713,752 cases and 17,539 hospitalizations, 2.0% fewer than our main estimates for the same scenario. The similarity of these results to those presented in the main text suggest our simplified fitting approach generates estimates of

© 2022 Rainisch G et al. *JAMA Network Open*.

averted cases that are sufficiently accurate for policymakers to value the impact of CICT, while preserving a simple, easy-to-use model for public health practitioners.

© 2022 Rainisch G et al. *JAMA Network Open*.

eFigure 5. Fitted Epidemic Curve Outputs From COVIDTracer Advanced and Observed Data for the 60-Day Period, by Jurisdiction and Fitting Approach

(Case counts excluded to maintain jurisdiction anonymity)

<u>Jurisdiction 1</u>

Fits Using Cumulative Cases

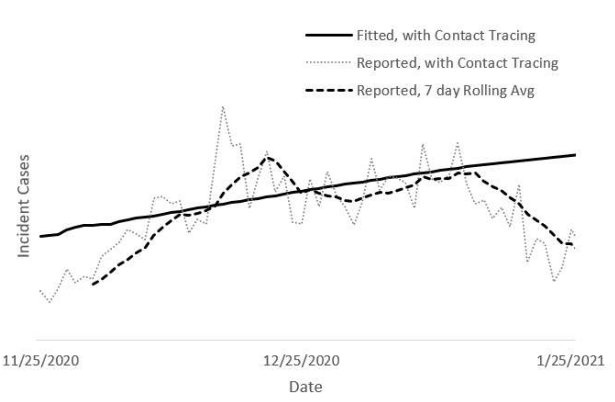

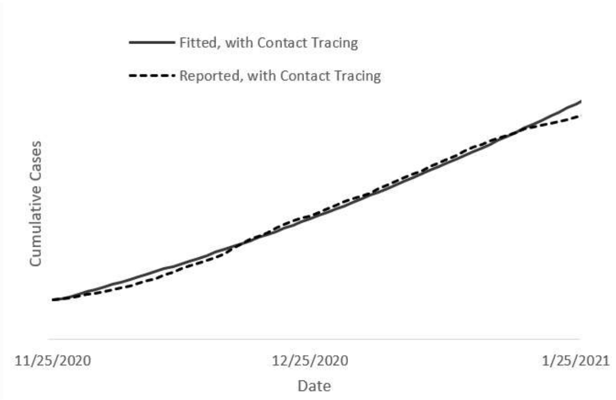

Fits Using Incident Cases' 7-day Moving Average and Multiple Fitting Periods

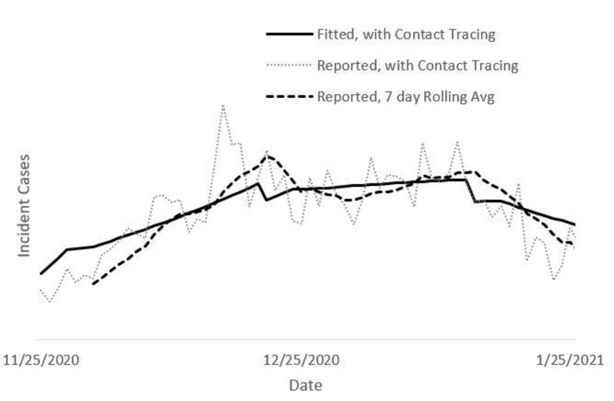

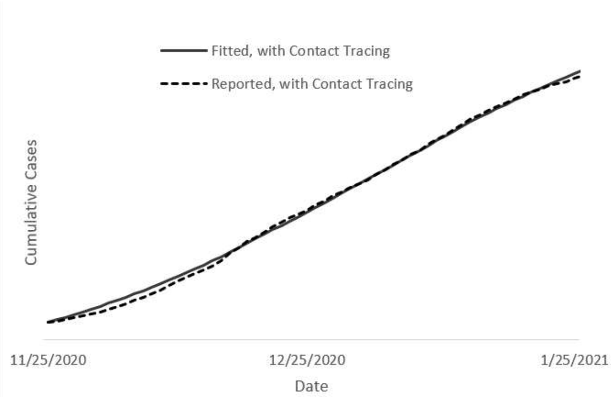

© 2022 Rainisch G et al. *JAMA Network Open*.

Jurisdiction 2

Fits Using Cumulative Cases

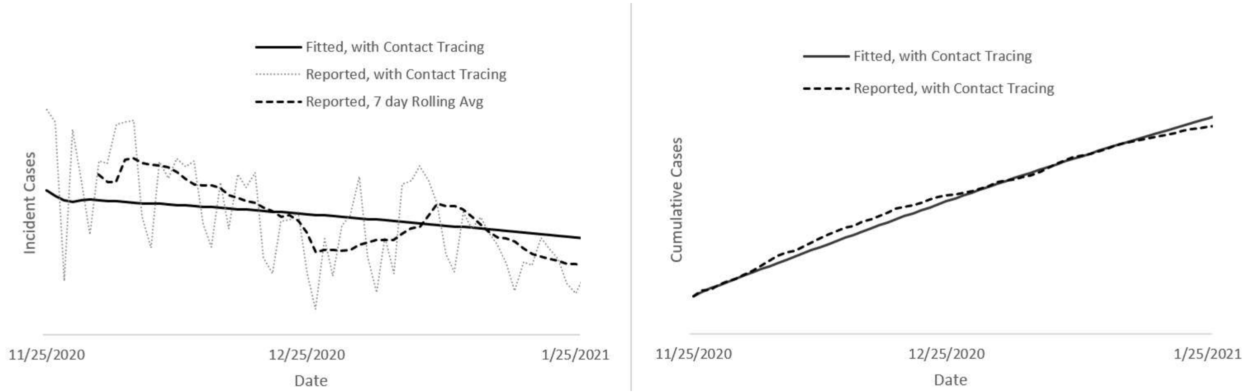

Fits Using Incident Cases’ 7-day Moving Average and Multiple Fitting Periods

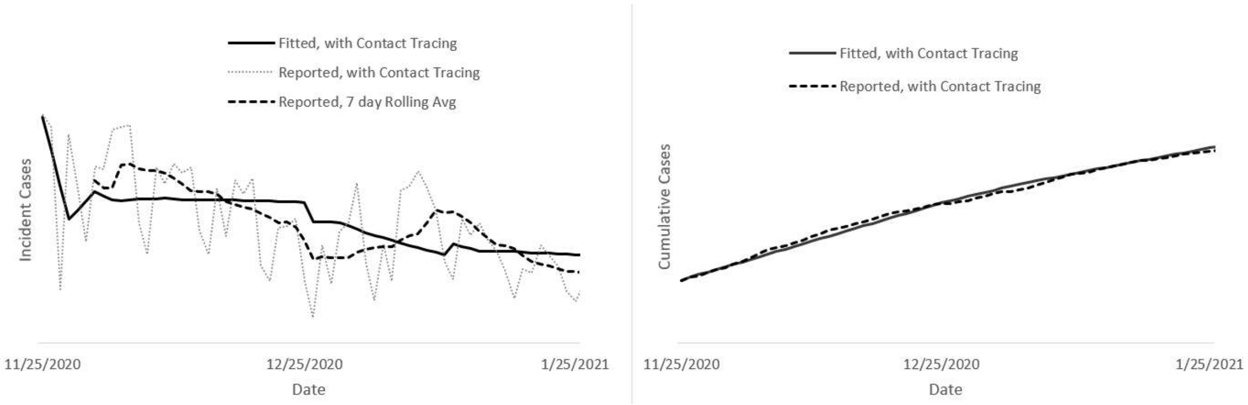

© 2022 Rainisch G et al. *JAMA Network Open*.

Jurisdiction 3

Fits Using Cumulative Cases

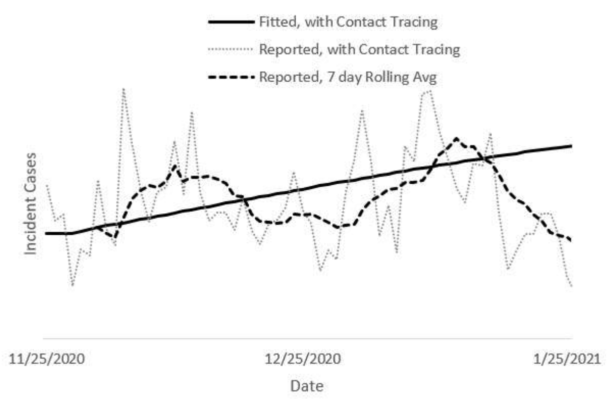

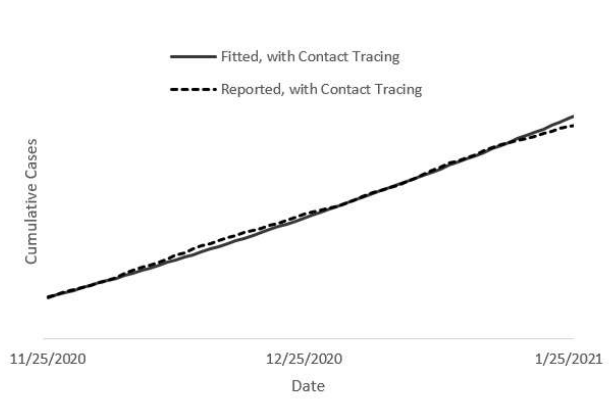

Fits Using Incident Cases' 7-day Moving Average and Multiple Fitting Periods

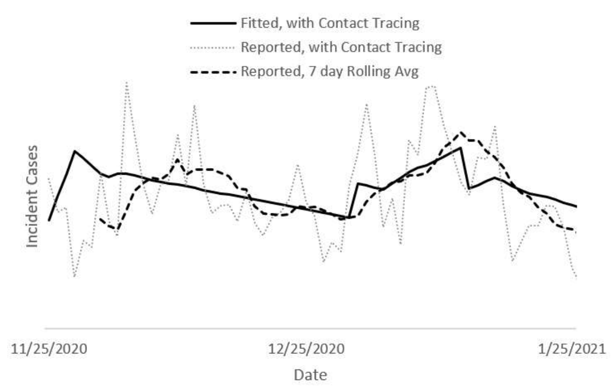

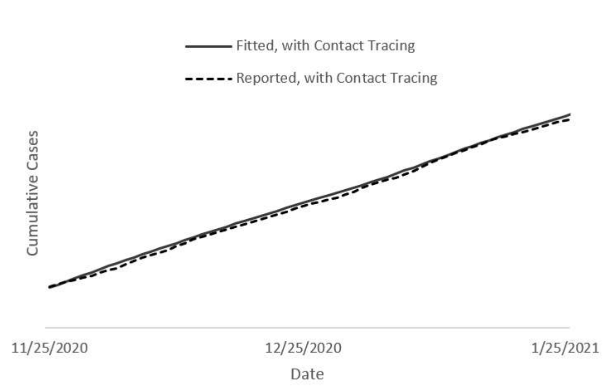

© 2022 Rainisch G et al. *JAMA Network Open*.

Jurisdiction 4

Fits Using Cumulative Cases

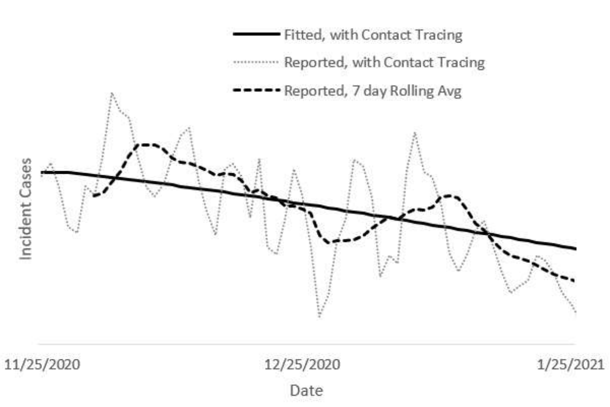

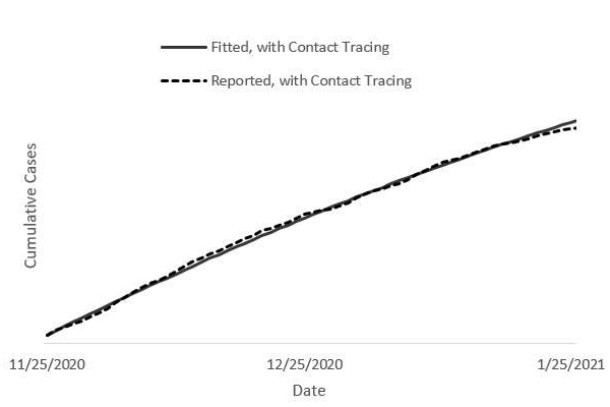

Fits Using Incident Cases' 7-day Moving Average and Multiple Fitting Periods

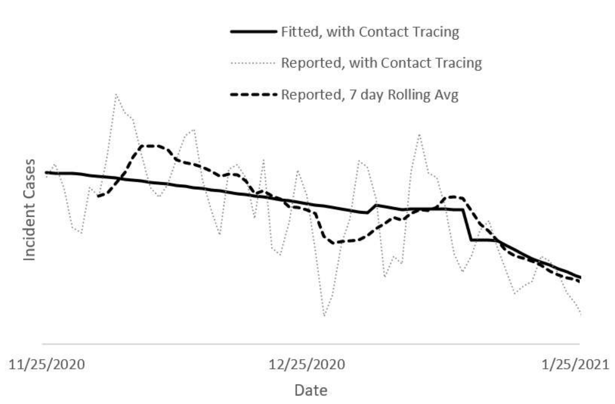

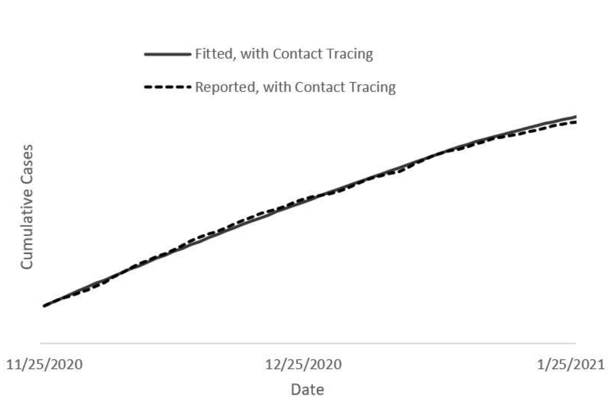

© 2022 Rainisch G et al. *JAMA Network Open*.

Jurisdiction 5

Fits Using Cumulative Cases

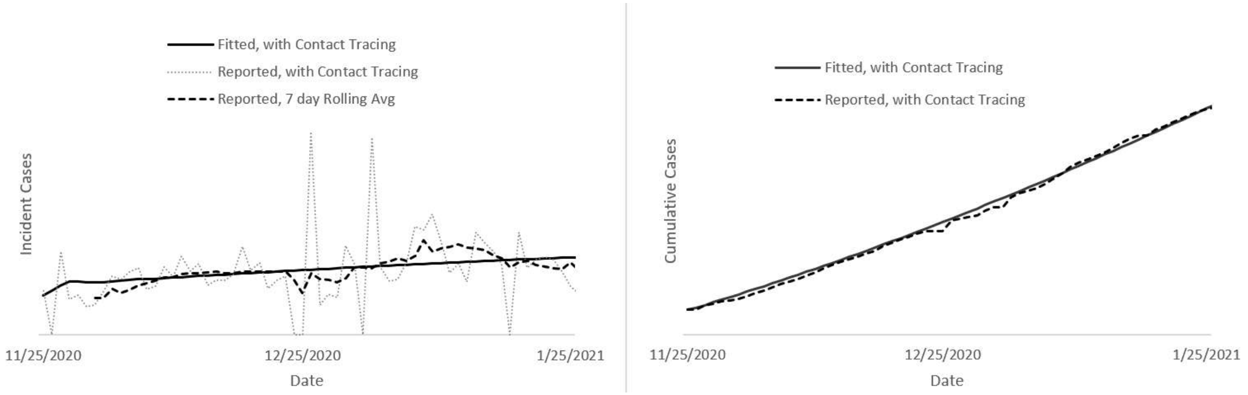

Fits Using Incident Cases' 7-day Moving Average and Multiple Fitting Periods*

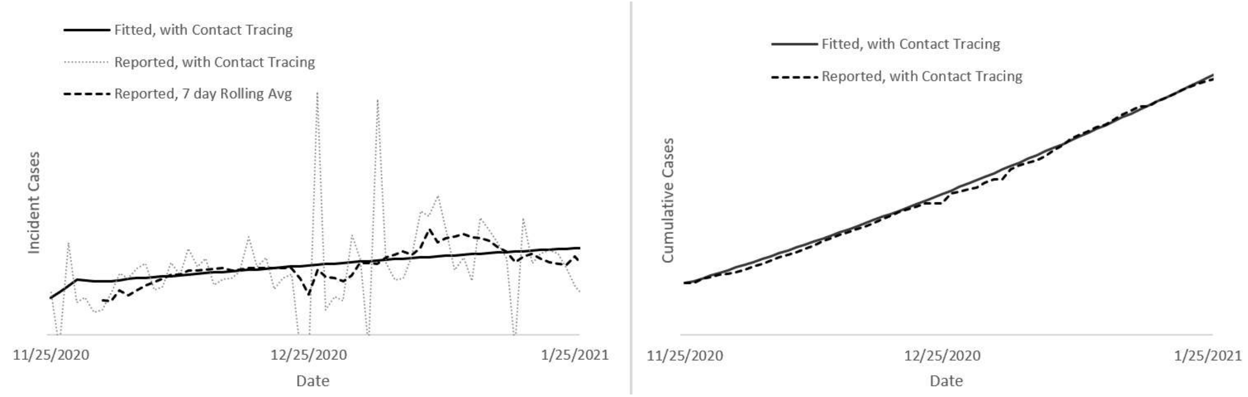

* A single fitting period spanning the entire 60-day evaluation period maximized fit for this jurisdiction.

© 2022 Rainisch G et al. *JAMA Network Open*.

Jurisdiction 7

Fits Using Cumulative Cases

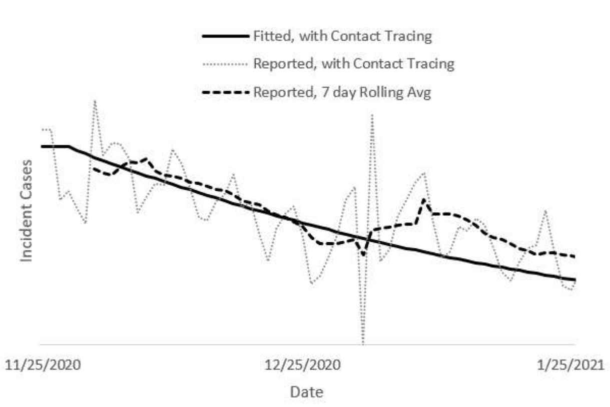

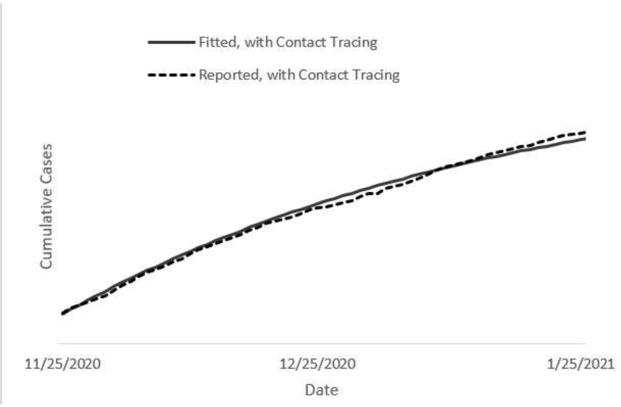

Fits Using Incident Cases' 7-day Moving Average and Multiple Fitting Periods*

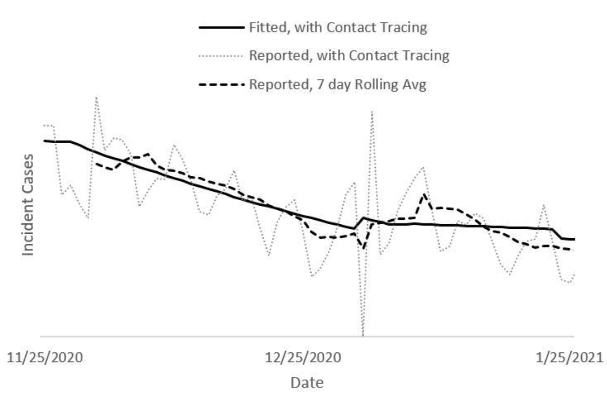

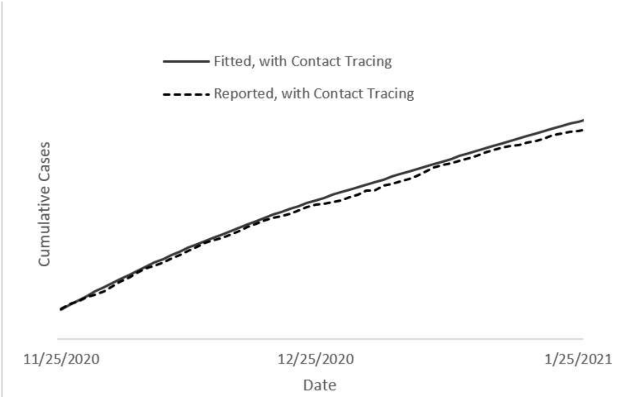

* Two fitting periods maximized fit for this jurisdiction.

© 2022 Rainisch G et al. *JAMA Network Open*.

<u>**Jurisdiction 12**</u>

Fits Using Cumulative Cases

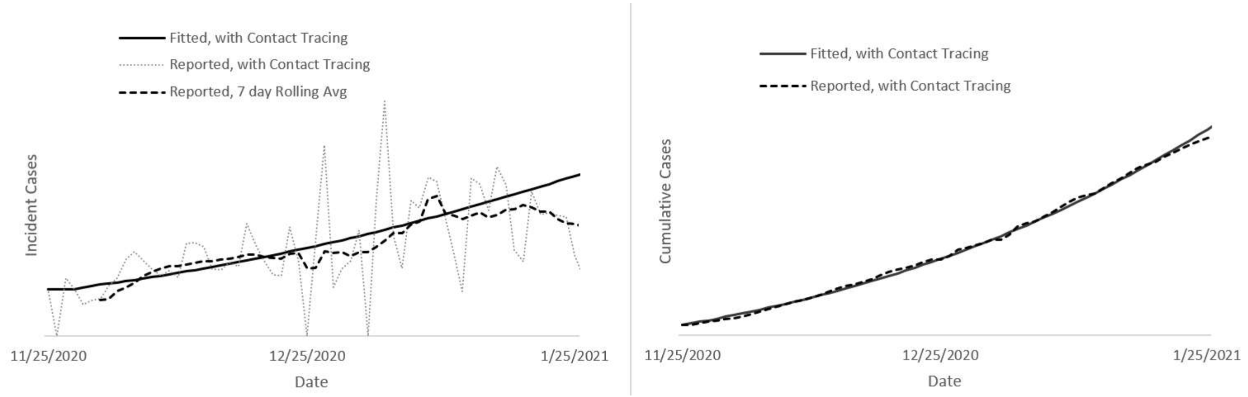

Fits Using Incident Cases' 7-day Moving Average and Multiple Fitting Periods

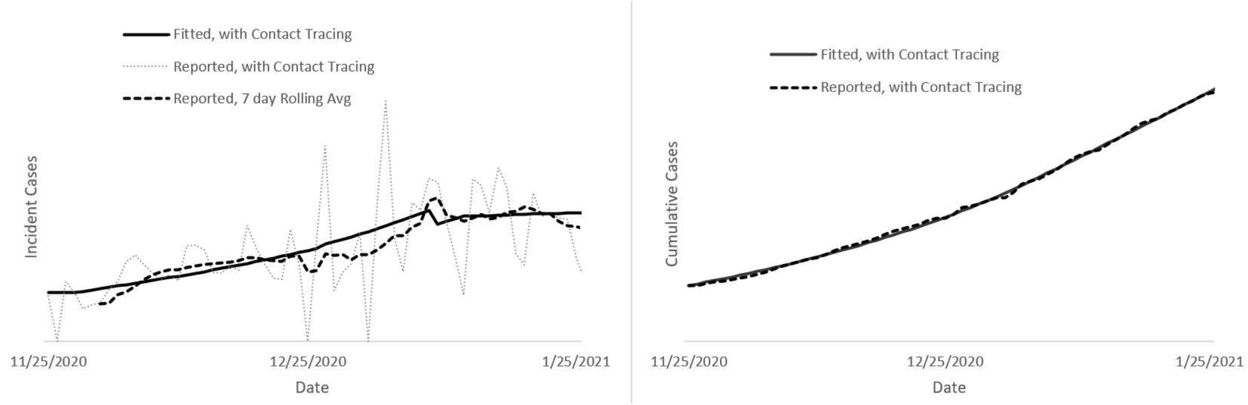

© 2022 Rainisch G et al. *JAMA Network Open*.

Jurisdiction 20

Fits Using Cumulative Cases

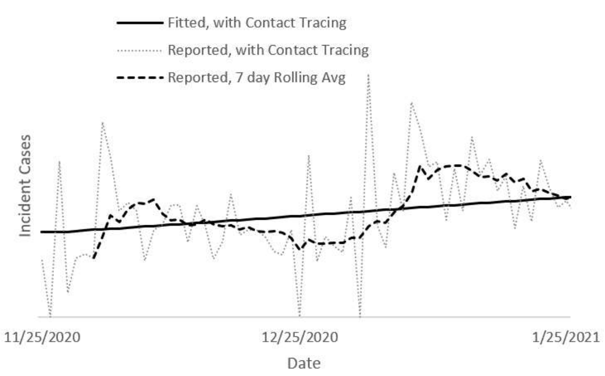

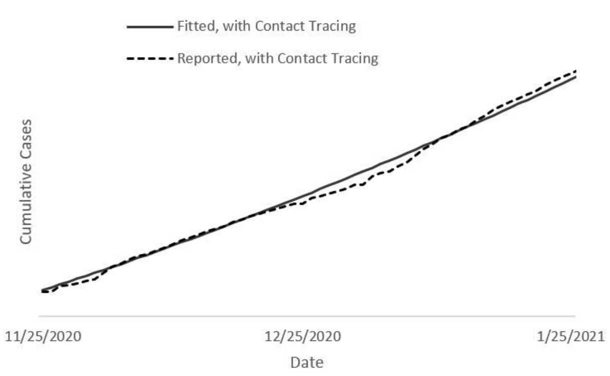

Fits Using Incident Cases' 7-day Moving Average and Multiple Fitting Periods

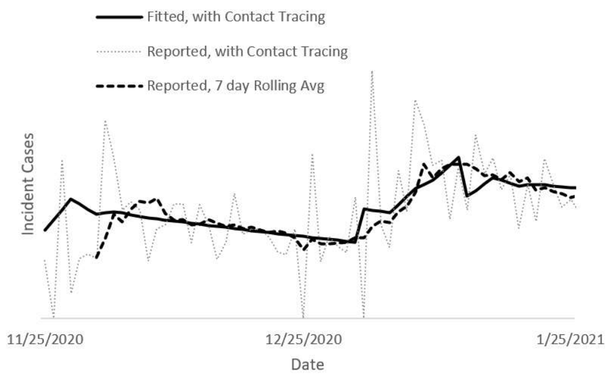

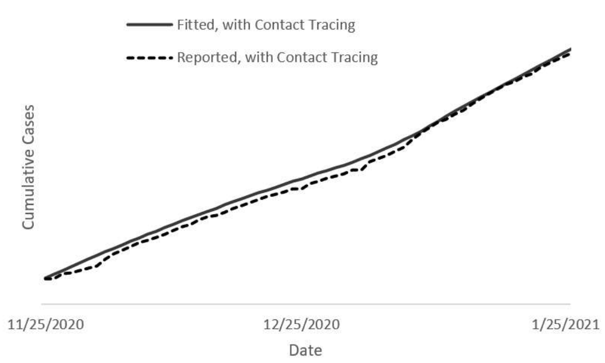

© 2022 Rainisch G et al. *JAMA Network Open*.

eTable 6. Effect of Alternate Fitting Methods[a] on Estimates of the Impact of Case Investigation and Contact Tracing (CICT) from 11/25/20 to 1/23/21 (60 Days), Under "Low" CICT Impact Scenario[b]

Jurisdiction	% Transmission reduction from Other NPIs & Vaccine[d]		Cases Averted by CICT[f], 60 days		Hospitalizations Averted by CICT[f], 60 days		% Reduction in cases and hospitalizations by CICT[g], 60 days	
	Main Results (Single, Constant NPI Effectiveness)	Alternate Fit (Up to three NPI Effectiveness Values[h])	Main Results	Results, Alternate Fit	Main Results	Results, Alternate Fit	Main Results	Results, Alternate Fit
**1[c]**	53.6	48.8, 55.2, 61.4	207,417	202,236	5,097	4,970	12.8	13.0
**2**	53.6	52.6, 59.1, 52.9	121,865	113,234	2,995	2,782	37.5	36.3
**3**	51.6	55.9, 47.9, 56.5	120,157	107,950	2,953	2,653	42.6	40.4
**4**	56.5	56.5, 53.9, 62.2	97,231	96,477	2,389	2,371	23.8	23.6
**5**	54.1	54.1	70,297	71,027	1,727	1,745	15.8	15.8
**7[c]**	59.7	59.6, 54.1	73,780	85,831	1,813	2,109	22.2	23.3
**12**	50.1	50.0, 48.4, 54.9	24,011	22,823	590	561	10.0	9.8
**20**	49.3	54.8, 34.4, 52.2	13,560	14,080	333	346	65.8	65.6

[a] Using the 7-day moving incidence average for fitting and up to three periods for each jurisdiction using our low CICT impact scenario (Table 1).
[b] Low impact scenario assumes 80% of actively monitored contacts (who later became cases), 30% of notified contacts effectively quarantined/isolated and 80% interviewed cases fully adhered to isolation guidelines.
[c] Single large city or county in these states were separate Epidemiology and Laboratory Capacity (ELC) jurisdictions and not included in this analysis.
[d] Percent reduction in the number of new infections per case (R_t) due to a combination of vaccination and all other nonpharmaceutical interventions (NPIs; *e.g.,* masks use, social distancing, school/restaurant closures, etc). Calculated as the percent difference in R_0 and R_t after implementation of vaccine and other NPIs.
[e] Percent reduction in the number of new infections per case (R_t) due to CICT after the implementation of other NPIs. Calculated as the percent difference between R_t after implementation of other NPIs and R_t after implementation of both other NPIs and CICT.
[f] After accounting for the impacts from vaccination and all other NPIs.
[g] Cases or hospitalizations averted by CICT out of the estimated cases or hospitalizations remaining after the implementation of vaccination and other NPIs.
[h] See eFigure 5 for fitted curves and periods used for fitting.

© 2022 Rainisch G et al. *JAMA Network Open*.

eReferences

1. Adhikari BB, Arifkhanova A, Coronado F, et al. COVIDTracer Advanced: A Planning Tool to Illustrate the Resources Needed to Conduct Contact Tracing and Monitoring of coronavirus disease 2019 (COVID-19) Cases and the Potential Impact of Community Interventions and Contact Tracing Efforts on the Spread of COVID-19 https://www.cdc.gov/coronavirus/2019-ncov/php/contact-tracing/COVIDTracerTools.html
2. He X, Lau EHY, Wu P, et al. Temporal dynamics in viral shedding and transmissibility of COVID-19. *Nature Medicine*. 2020;26(5):672-675. doi:10.1038/s41591-020-0869-5
3. He X, Lau EHY, Wu P, et al. Author Correction: Temporal dynamics in viral shedding and transmissibility of COVID-19. *Nature Medicine*. 2020;26(9):1491-1493. doi:10.1038/s41591-020-1016-z
4. Ferretti L, Wymant C, Kendall M, et al. Quantifying SARS-CoV-2 transmission suggests epidemic control with digital contact tracing. *Science*. 2020;368(6491):eabb6936. doi:10.1126/science.abb6936
5. CDC. COVID-19 Pandemic Planning Scenarios. Aug 5, 2021. Updated Mar 19, 2021. https://www.cdc.gov/coronavirus/2019-ncov/hcp/planning-scenarios.html
6. CDC. COVID Data Tracker. https://covid.cdc.gov/covid-data-tracker/
7. CDC COVID-19 Response Team. Severe Outcomes Among Patients with Coronavirus Disease 2019 (COVID-19) — United States, February 12–March 16, 2020. *Morbidity and Mortality Weekly Report*. 2020;69(12):343-346.
8. Wu SL, Mertens AN, Crider YS, et al. Substantial underestimation of SARS-CoV-2 infection in the United States. *Nature Communications*. 2020;11(4507)doi:https://doi.org/10.1038/s41467-020-18272-4
9. CDC. Interim Guidance on Ending Isolation and Precautions for Adults with COVID-19. https://www.cdc.gov/coronavirus/2019-ncov/hcp/duration-isolation.html
10. Jeon S, Rainisch G, Lash RR, et al. Estimates of cases and hospitalizations averted by COVID-19 case investigation and contact tracing in 14 health jurisdictions in the United States. *Journal of Public Health Management and Practice*. 2021;In Press
11. Smith LE, Potts HWW, Amlôt R, Fear NT, Michie S, Rubin GJ. Adherence to the test, trace, and isolate system in the UK: results from 37 nationally representative surveys. *BMJ*. 2021;372:n608. doi:https://doi.org/10.1136/bmj.n608
12. Park CL, Russell BS, Fendrich M, Finkelstein-Fox L, Hutchison M, Becker J. Americans' COVID-19 Stress, Coping, and Adherence to CDC Guidelines. *Journal of General Internal Medicine*. 2020;35:2296–2303.
13. *The Challenges of Contact Tracing as U.S. Battles COVID-19*. 2020. October. https://www.pewresearch.org/internet/2020/10/30/the-challenges-of-contact-tracing-as-u-s-battles-covid-19/

© 2022 Rainisch G et al. *JAMA Network Open*.
